# Solvent and Ion‐Mediated Behavior of a Thermoresponsive Brush: Specific Ion Effects in Methanol‐Water Electrolytes

**DOI:** 10.1002/marc.202500093

**Published:** 2025-05-13

**Authors:** Hayden Robertson, Joshua D. Willott, Andrew R.J. Nelson, Stuart W. Prescott, Erica J. Wanless, Grant, B. Webber

**Affiliations:** ^1^ Soft Matter at Interfaces, Institute for Condensed Matter Physics Technical University of Darmstadt D‐64289 Darmstadt Germany; ^2^ College of Science Engineering and Environment University of Newcastle Callaghan NSW 2308 Australia; ^3^ Australian Centre for Neutron Scattering ANSTO Locked bag 2001 Kirrawee DC NSW 2232 Australia; ^4^ School of Chemical Engineering UNSW Sydney Sydney NSW 2052 Australia

**Keywords:** cononsolvency, ellipsometry, neutron reflectometry, PNIPAM, polymer brush, specific ion effects

## Abstract

In this study, specific ion effects are explored in methanol‐water mixtures, which play a critical role in a diverse range of applications, including protein solubilization and supercapacitors. Spectroscopic ellipsometry and neutron reflectometry are employed to investigate the solvent‐ and ion‐mediated behavior of a poly(*N*‐isopropylacrylamide) (PNIPAM) brush, a well‐known thermoresponsive polymer. In the absence of ions and at low methanol mole fractions (*x*_M_), PNIPAM displays lower critical solution temperature (LCST) type behavior, with the thermotransition temperature decreasing as *x*_M_ increased. Upon further increasing *x*_M_, a cononsolvency region is identified at approximately *x*_M_ = 0.15, beyond which re‐entrant swelling is observed in conjunction with a suppressed thermoresponse. In the presence of *x*_M_ = 0.10 electrolytes, the observed specific ion effects adhere to a *forward* Hofmeister series. Strongly solvated ions, such as Cl^–^ and Br^–^, decrease the LCST of the brush. In contrast, poorly solvated ions, such as SCN^–^ and I^–^, lead to more swollen brush profiles and an increase in the LCST. We hypothesize that the stability of water‐methanol clusters plays a crucial role in governing polymer solvation, providing insights into the fundamental interactions within mixed solvent systems. Moreover, a theoretical ion that does not impact the swelling or structure of a PNIPAM brush is proposed.

## Graphical abstract

The manifestation of specific ion effects in methanol‐water mixtures on polymer brush exemplars is explored. Spectroscopic ellipsometry and neutron reflectometry revealed solvent‐driven LCST changes in a thermoresponsive brush, which is also readily influenced by the presence of electrolytes. Effects are observed to follow a Hofmeister series, and a theoretical ion imposing a “null” impact on the system is also proposed.

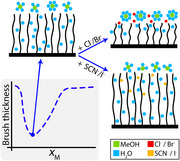

## 1 Introduction

Polymer brushes consist of dense arrays of surface‐tethered polymer chains. When composed of stimuli‐responsive polymers, these brushes can exhibit switchable surface properties, such as adhesion and lubrication, in response to external stimuli like temperature and pH.^[^ ^1^, ^2^, ^3^, ^4^, ^5^ ^]^ One such polymer is poly(*N–*isopropylacrylamide) (PNIPAM), a thermoresponsive polymer that transitions from a well‐solvated to a poorly‐solvated state with increasing temperature.^[^ ^6^, ^7^ ^]^ The point of this transition is commonly referred to as the lower critical solution temperature (LCST).^[^ ^8^, ^9^ ^]^ For a PNIPAM brush, this change in solvation state manifests as a swollen to collapsed transition. Polymer brushes serve as an exemplar system to probe further physicochemical phenomena as polymer concentration remains invariant; thus polymer swelling and nanostructure can be examined regardless of the polymer solvation.

**Figure 1:**
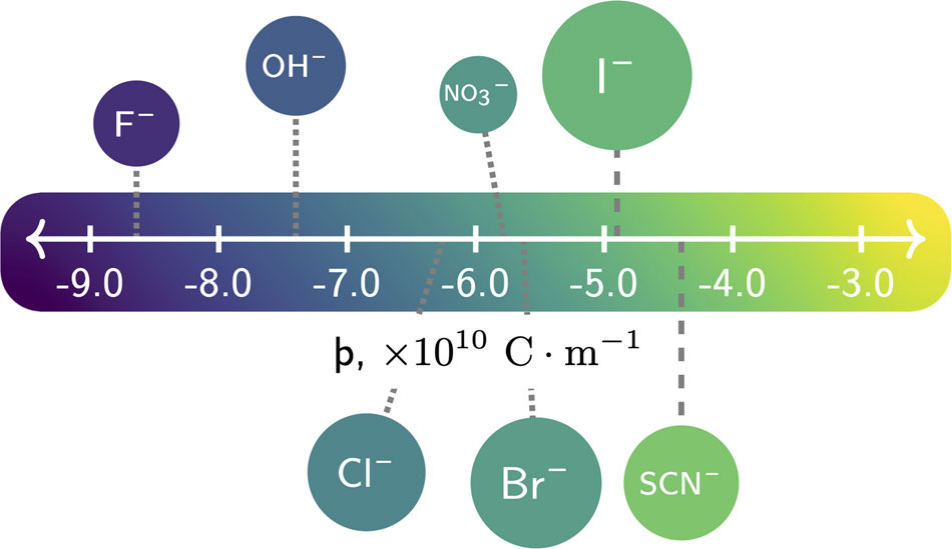
þ values are presented for a selection of anions from the Hofmeister series. Anion color and position indicate the þ value and the size depicted is proportional to $\sqrt[{3}]{r_{\mathrm{ion}}^{3}}$, where $r_{\mathrm{ion}}$ is the radial moment of the ion. All anions are presented as spheres for representation. þ values are calculated in a vacuum and are independent of solvent composition. þ and $\sqrt[{3}]{r_{\mathrm{ion}}^{3}}$ values are taken from Gregory et al.^[^ ^23^ ^]^

First noted by Franz Hofmeister, specific ion effects (SIE) pertain to any phenomena resulting from ion identity rather than concentration or valency.^[^ ^10^, ^11^ ^]^ These effects are pervasive in both natural and industrial systems, influencing the stability of polymers,^[^ ^12^, ^13^, ^14^, ^15^, ^16^ ^]^ colloidal particles,^[^ ^17^, ^18^ ^]^ and processes like froth flotation and mineral extraction.^[^ ^19^, ^20^ ^]^ The qualitative Hofmeister series ranks ions according to their ability to stabilize (salt‐in) or destabilize (salt‐out) macromolecules in solution: ions on the *left* of the series as depicted in **Figure** **1**, such as Cl^−^, destabilize macromolecules, while those on the *right*, such as SCN^−^, promote stabilization. In particular, the effects imparted by ions are highly system dependent, where central ions, such as Br^−^, have the ability to translate between manifesting salting‐in and salting‐out behavior.^[^ ^21^ ^]^ However, for the instance of the PNIPAM brush systems, Br^−^ is known to be salting‐out anion.^[^ ^22^ ^]^ Historically, the effects of ions were primarily attributed to their influence on water structure, classifying ions as either kosmotropic or chaotropic. However, contemporary computational and experimental studies have revealed that fully understanding the impact of ions requires considering the numerous complex interactions among the solvent, solute, and substrate. In a recent study, Gregory et al. quantitatively described SIE in terms of the interaction site‐specific radial charge density of an ion: þ (“sho”).^23^ Figure 1 presents common anions and their associated þ values: classically salting‐out anions (blue) exhibit more negative þ values compared to salting‐in anions (green).

The behavior of both free and grafted PNIPAM has been well‐studied in aqueous solution, making it a suitable choice for comparison in non‐aqueous electrolyte solutions.^[^ ^13^, ^14^, ^24^, ^25^ ^]^ Our previous studies examined the change in swelling and nanostructure of a PNIPAM brush in various potassium and lithium aqueous electrolytes, noting the expected salting‐in (e.g., SCN^−^) and salting‐out (e.g., Cl^−^) behavior.^[^ ^22^ ^]^ The anion was deduced to modify the “type” of response (i.e., salting‐in or salting‐out) whereas the cation was seen to merely modulate the magnitude of the effect. The impact of the anion on the LCST of the brush was strongly correlated to the ion's þ value. The results from this study are reproduced in Figure S3.1 (Supporting Information).

Despite the importance of advancing our understanding of SIE, their behavior in non‐aqueous solvents remains largely unexplored. Methanol, a versatile amphiphilic solvent, is pivotal in numerous chemical reactions and industrial applications. The swelling behavior of PNIPAM in methanol has been previously investigated, with both pure water and pure methanol recognized as “good” solvents for PNIPAM.^[^ ^26^, ^27^, ^28^ ^]^ Liu et al. have noted that under isothermal conditions and in solvent mixtures of water and methanol, PNIPAM exhibits cononsolvency behavior at intermediate mole fractions of methanol (*x*_M_).^[^ ^26^ ^]^ In these water‐methanol mixtures, solvent aggregates form, where their composition is a function of the overall *x*_M_.^[^ ^26^, ^29^, ^30^, ^31^, ^32^ ^]^ The presence of solvent aggregates aids cooperative dehydration, limiting both the formation of a microscopically continuous solvent phase as well as the hydrogen‐bonding sites available to solvate PNIPAM.^[^ ^26^, ^33^, ^34^, ^35^, ^36^ ^]^ This is due to the strength of the water‐methanol hydrogen bonding interaction being almost equivalent to that of water‐water and methanol‐methanol.^[^ ^37^ ^]^ Quasi‐elastic neutron scattering measurements by Kyriakos et al. have identified two populations of water in ternary mixtures of water, methanol, and PNIPAM: “strongly arrested” and “less‐strongly arrested” water.^[^ ^30^ ^]^ Upon surpassing the LCST, the strongly arrested water is only partially released from PNIPAM, meaning PNIPAM is never fully desolvated. However, the role of methanol in modulating SIE within polymer systems and mixed solvents remains largely under‐explored, requiring further investigation to unravel the underlying mechanisms and expand its practical applications. It is expected, however, that SIE will manifest differently in non‐aqueous solvents and their mixtures with water due to the formation of solvent clusters.

A key area of interest is understanding how the addition of ions influences polymer conformation and nanostructure in mixed solvents, particularly in water‐methanol systems. Herein, for the first time, we probe the solvent and ion‐modulated conformation of a PNIPAM brush as a function of temperature in water‐methanol systems. The choice of ions was informed by previous investigations, ensuring solubility across a broad range of solvents whilst also representing a broad range of þ values (see Figure 1).^[^ ^22^, ^23^ ^]^ Understanding SIE in complex environments (e.g., methanol electrolytes) is crucial for future applications, including pharmaceuticals and controlling polymer and biomolecular conformations for targeted drug delivery.

## 2 Results and Discussion

The swelling and structure of PNIPAM brushes were monitored as a function of both electrolyte identity and solvent composition. Initially, the baseline behavior of the brush was characterized by spectroscopic ellipsometry (SE), in the absence of salt, across water‐methanol solvents with varying methanol mole fractions (*x*_M_). This was complemented by neutron reflectivity (NR) derived polymer volume fraction (VF) profiles of the PNIPAM brush in *x*_M_ = 0.10 as a function of temperature. The impact of ions on the brush thermoresponse and structure was then examined with both SE and NR in water‐methanol solvent composed of *x*_M_ = 0.10. Electrolyte concentrations are reported in mol% to maintain a consistent solute/solvent molecule ratio and were selected to be 0.2 mol% and 0.9 mol% (≈100 and 500 mMm in water, respectively).

### 2.1 Impact of Pure Solvents on Brush Swelling and Structure

**Figure 2:**
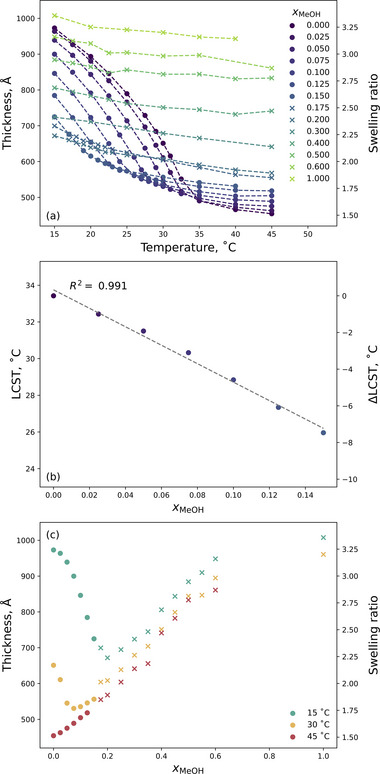
Ellipsometrically determined brush thickness of a 300 Å PNIPAM brush as a function of both temperature and solvent composition. Selected *x*_M_ are shown in (a), whereas (c) presents selected temperatures. (b) Extracted brush LCST as a function of *x*_M_. Circle symbols (•) denote regions where a clear LCST is exhibited whereas crosses (×) denote compositions that yield a suppressed thermoresponse. Uncertainties are smaller than the markers.

Prior to the addition of salt, spectroscopic ellipsometry was conducted on a brush of 300 Å dry thickness (see **Table** **1**) to establish baseline behavior in various binary water‐methanol solvents. **Figure** **2** presents the ellipsometrically derived solvated brush thickness and swelling ratio as a function of both temperature and *x*_M_. Here data are grouped into two regimes: solvents in which PNIPAM exhibits LCST‐type behavior (•) and solvents in which no clear thermotransition is observed (×) across the examined temperature range (Figure 2a). The corresponding derived LCST values of the brush, where observed, are also presented (Figure 2b).

**Table 1:** Summary of PNIPAM dry brush thickness^a)^, determined via air‐solid ellipsometry and neutron reflectometry for each brush sample studied during designated in situ measurements.

Sample examined during specified in situ measurement	Ellipsometrically determined dry brush thickness*^b^ *^)^, Å	NR determined dry brush thickness, Å
Ellipsometry (no salt)	300 ± 1	–
Ellipsometry (salt)	322 ± 1	–
NR	210 ± 5	220 ± 2

^a)^ Associated uncertainties for ellipsometry measurements are taken as the standard deviation from multiple measurements across the surface. For neutron reflectometry measurements, uncertainties are derived from PT‐MCMC sampling; see details in the *Neutron reflectometry analysis* section.

^b)^ The ellipsometrically determined dry‐brush thickness is used as the sample identifier for experiments.

PNIPAM is known to exhibit LCST‐type behavior in pure water (*x*_M_ = 0), undergoing a clear collapse transition at ≈33 °C.^[^ ^6^, ^38^ ^]^ Moreover, pure methanol (*x*_M_ = 1.0) is known to be a much better solvent for PNIPAM than water.^[^ ^39^ ^]^ In both these pure solvents, water‐PNIPAM (at low temperatures) and methanol‐PNIPAM hydrogen bonds are more favorable than polymer self‐interactions, keeping PNIPAM solvated and swollen.^[^ ^26^, ^40^ ^]^ However, upon exposure to water‐methanol mixtures, the behavior of the brush is governed by the formation of solvent complexes; water‐methanol hydrogen bonding is far stronger than water‐PNIPAM interactions.^[^ ^26^, ^41^ ^]^

At very low *x*_M_ (i.e., water‐rich environments), the brush thickness at a given temperature (Figure 2a), and the LCST (Figure 2b), decrease with increasing *x*_M_. This decrease in PNIPAM LCST with increasing *x*_M_ is observed here up to approximately *x*_M_ = 0.15 and is concordant with studies probing untethered PNIPAM in water‐methanol binary solvents,^[^ ^29^, ^42^, ^43^ ^]^ PNIPAM microgels in water‐methanol binary solvents,^[^ ^35^, ^39^ ^]^ as well as analogous behavior observed for PNIPAM brushes in water‐DMSO systems.^[^ ^22^, ^38^ ^]^ In this regime, because water molecules are responsible for the solvation of PNIPAM, the addition of methanol decreases the LCST of PNIPAM; methanol acts as a kosmotrope, dehydrating the polymer in favor of water‐methanol complexation.^[^ ^26^, ^34^ ^]^ In particular, the decrease in polymer solvation arises from the decrease in the available solvent hydrogen bonding sites as a result of solvent complexation.

Upon slightly increasing the *x*_M_ further, a cononsolvency region is achieved,^[^ ^44^ ^]^ whereby competitive hydrogen bonding limits polymer solvation;^[^ ^29^, ^45^ ^]^ sufficient methanol is present within the system to occupy free‐water molecules in the preferred water‐methanol complexation. It is at these intermediate *x*_M_ compositions that the brush is in its most collapsed state across the entire temperature range examined, as water‐methanol complexes are poor solvents for PNIPAM.^[^ ^31^ ^]^ Further increasing *x*_M_ reveals re‐entrant swelling in conjunction with a suppressed thermoresponse. This can be attributed to the cooperative solvation of the brush periphery by unbound methanol molecules as well as methanol and water‐methanol complexes bridging polymer chains;^[^ ^26^, ^35^ ^]^ PNIPAM‐methanol hydrogen bonds are more favorable than PNIPAM‐water hydrogen bonds.^[^ ^39^ ^]^ Here the brush retains its structure across the examined temperature range as the change in temperature is insufficient to disrupt the bridging polymer‐methanol hydrogen bonds; i.e., the thermoresponse of the brush remains suppressed. The limited thermoresponse of PNIPAM in methanol‐rich solvent conditions aligns with previous microgel studies, that demonstrated the polymer exhibits very little to no temperature‐dependent behavior under these solvent conditions.^[^ ^39^, ^44^ ^]^ These changes in solvent structure are complemented by Fourier‐transform infrared (FTIR) spectroscopy data presented in Figure S2.1 (Supporting Information), which reveal a simultaneous increase in the C‐O stretch and a slight blue shift with increasing methanol content. Interestingly, across this methanol concentration region, whilst the temperature‐responsive nature of PNIPAM appears to be suppressed, brush swelling is still seen to increase with increasing *x*_M_.

**Figure 3:**
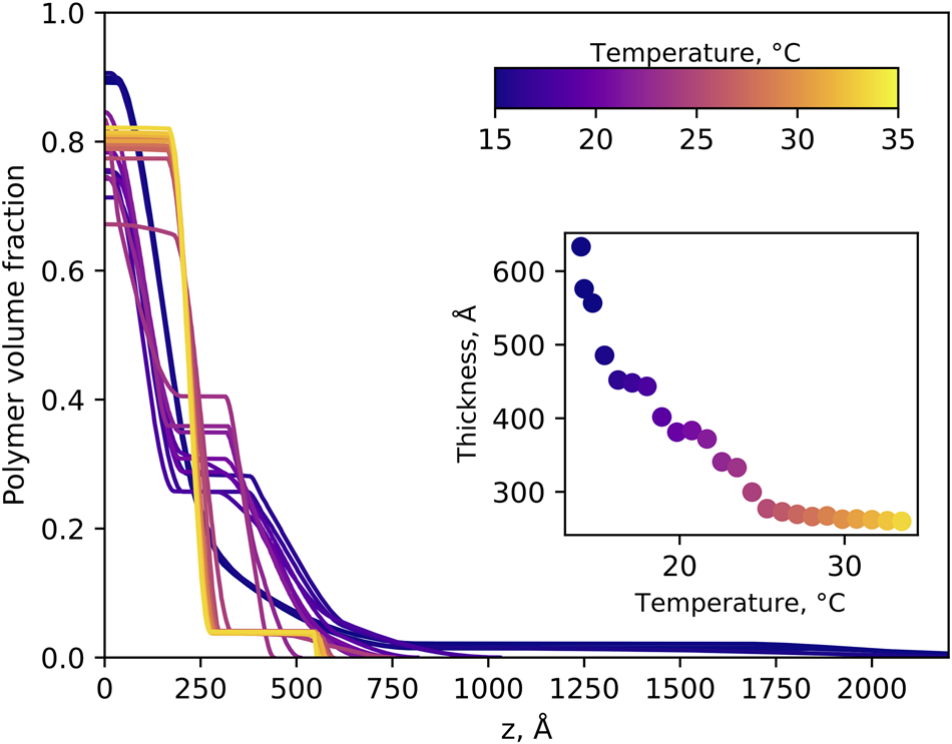
Polymer volume fraction profiles of a 210 Å PNIPAM brush in *x*_M_ = 0.10 as a function of temperature. Neutron reflectivity data were collected in event mode. Corresponding experimental and modeled reflectivities are presented in Figure S4.1 (Supporting Information).

Informed by the results in Figure 2, all subsequent measurements (including those with electrolytes) were conducted with *x*_M_ = 0.10 due to the accessible thermotransition. In order to unveil the internal structure of the brush in *x*_M_ = 0.10, a 210 Å dry thickness PNIPAM brush was examined with neutron reflectometry (Table 1). Here kinetic data were collected via event mode acquisition while the temperature of the measurement cell was changed, which enabled monitoring of the brush structure with a higher temporal, and thus thermal, resolution. These kinetic measurements provide an efficient overview of brush thickness and conformation across a broad temperature range, guiding the conditions of subsequent measurements. **Figure** **3** presents NR derived polymer volume fraction (VF) profiles, with the respective measured and modeled reflectivities presented in Figure S4.1 (Supporting Information). Kinetic NR data pertaining to the behavior of the PNIPAM brush in a broader suite of water‐methanol concentrations is presented in Figure S4.2 (Supporting Information) as well as the respective derived LCST values. As per our previous investigations, we refer to the polymer VF profile in terms of three regions: the “proximal layer” at the silicon/silica interface, an “inner region”, and a “diffuse tail” at the periphery of the brush.^[^ ^22^, ^38^, ^46^, ^47^ ^]^ At low temperatures, the brush is seen to be swollen: a small proximal layer with a very diffuse tail. However, upon increasing the temperature the inner region of the brush increases in polymer VF and the periphery of the brush becomes less diffuse as the tail collapses; the so‐called bottom‐up collapse.^[^ ^6^, ^48^ ^]^ At higher temperatures, the brush collapses to a more dense (i.e., “slab‐like”) film with a very well‐defined inner region and a more localized periphery. However, across all temperatures, the amount of polymer proximal to the interface remains relatively invariant.

### 2.2 Impact of Electrolytes on Brush Swelling and Structure

To probe the impact of methanol on the manifestation of specific ion effects, a 322 Å dry thickness PNIPAM brush (Table 1) was exposed to 0.2 and 0.9 mol% electrolytes composed of KSCN, KCl, LiI or LiBr in *x*_M_ = 0.10. The salts selected were chosen based on the solubilities in a wider suite of solvents examined as well as a wide range of þ values. The comparison behavior of a PNIPAM brush exposed to these electrolytes in water has been previously explored and is presented in Figure S3.1 (Supporting Information).^[^ ^22^ ^]^

**Figure 4:**
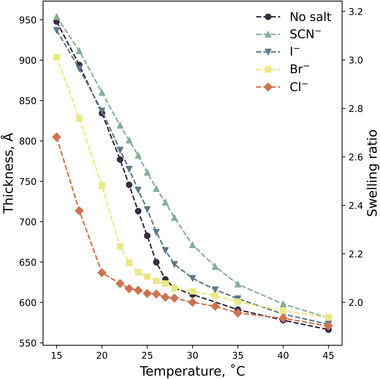
Ellipsometrically determined brush thickness (left) and swelling ratio (right) of a 322 Å PNIPAM brush exposed to 0.9 mol% KSCN, LiI, LiBr, and KCl electrolytes in water‐methanol composed of *x*_M_ = 0.10 as a function of temperature. Uncertainties are smaller than the markers.

As shown in Figures 2 and 3, a PNIPAM brush solvated by *x*_M_ = 0.10 undergoes an LCST‐type transition. Upon exposure to 0.9 mol% Br^−^, a classically salting‐out ion, the brush thickness at a given temperature is seen to decrease relative to the brush behavior in the absence of Br^−^ (**Figure** **4**). Similar behavior is also observed when exposing the brush to Cl−, another strongly solvated anion. However, the magnitude of the effect is greater, which is attributed to the higher charge density (þ) of Cl^−^. Conversely, upon exposure to SCN^−^ or I^−^, brush thickness is seen to increase relative to the absence of ions, demonstrating classic salting‐in behavior for these two ions. Here, SCN^−^ is observed to have the stronger salting‐in effect. For all electrolytes examined, the effects are analogous to those observed in water: a forward (or normal) Hofmeister series is discerned. Concordant results are presented in Figure S3.2 (Supporting Information) for equivalent 0.2 mol% electrolytes, demonstrating the same trend.

**Figure 5:**
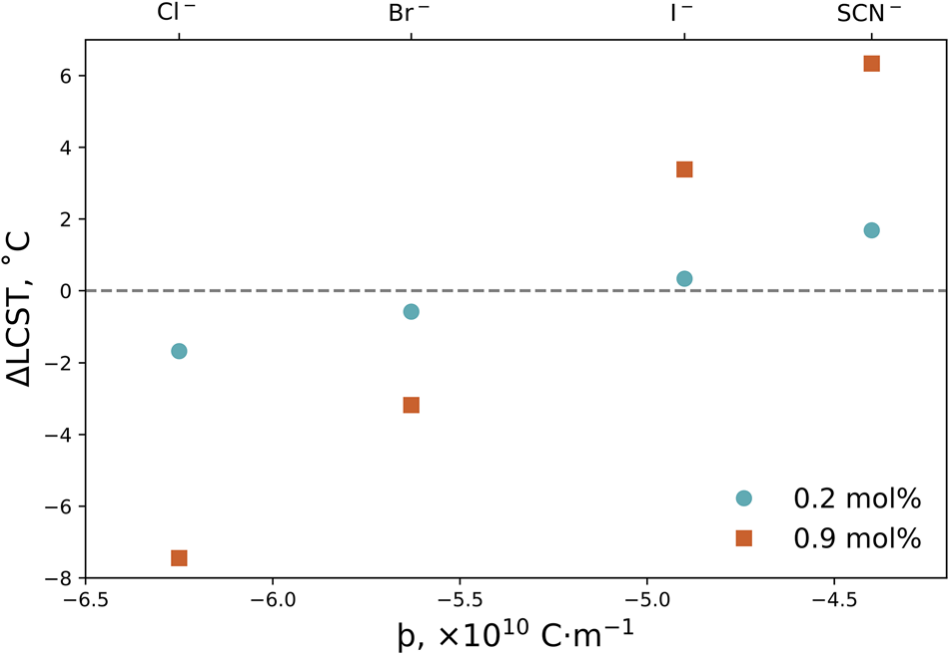
Change in lower critical solution temperature (∆LCST) of a PNIPAM brush in a water‐methanol solvent composed of *x*_M_ = 0.10 electrolytes (relative to the pure solvent) as a function of ion charge density, þ, and concentration. Top *x*‐axis illustrates the corresponding ion to the þ value.

The LCST can be extracted from Figure 4 by modeling the brush thickness with a logistic function (see Equation 1). **Figure** **5** presents the change in LCST of the 322 Å PNIPAM brush (relative to the pure solvent) as a function of electrolyte identity (i.e., þ). Corresponding þ values are presented in Table S1.1 (Supporting Information). Complementing the results from Figure 4, both Cl^−^ and Br^−^ yield a net decrease in LCST, whereas SCN^−^ and I^−^ result in a net increase in LCST. Here the magnitude of the effect is seen to increase with increasing electrolyte concentration. Both concentration series appear to follow a linear trend with ∆LCST and þ.

**Figure 6:**
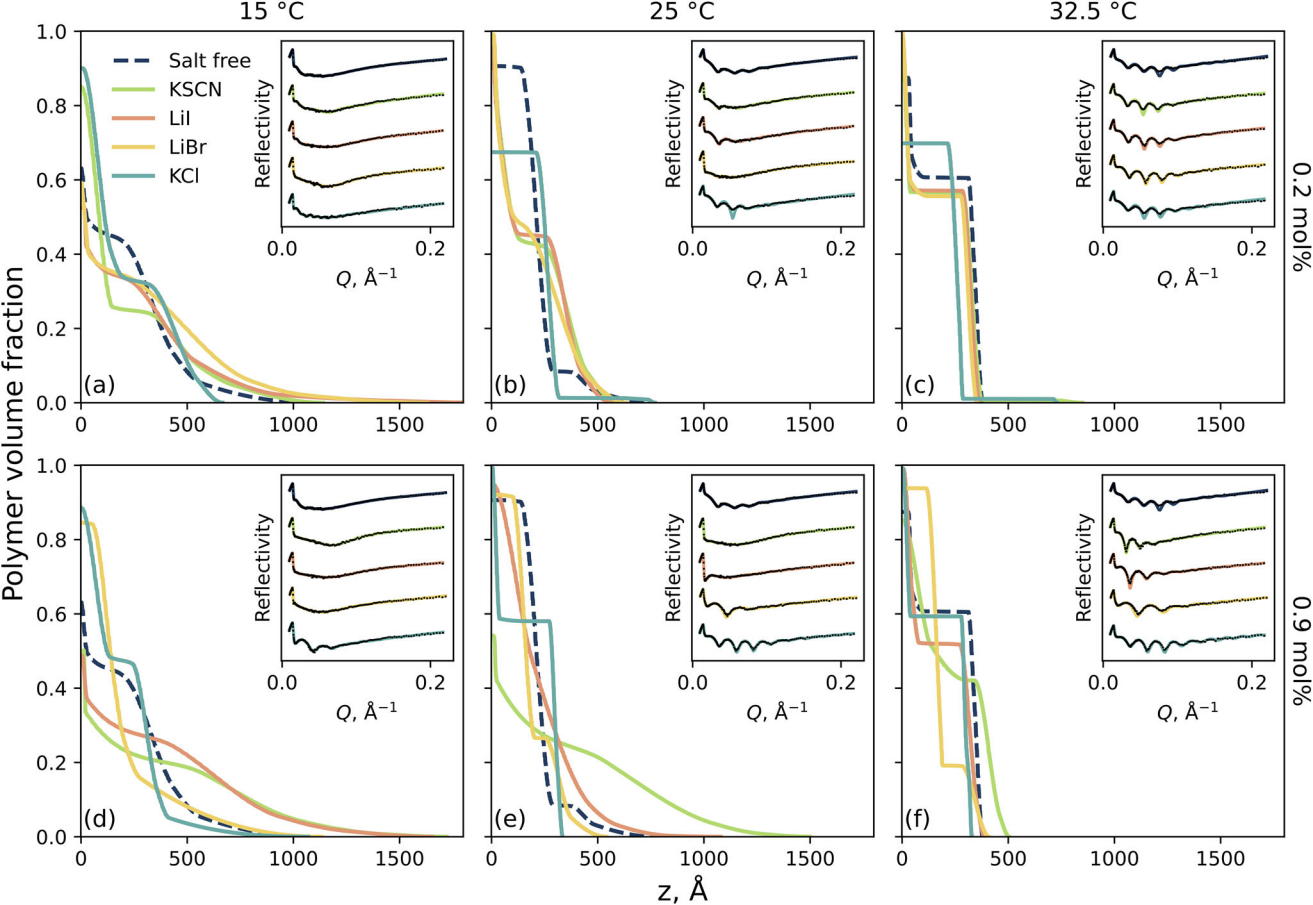
Neutron reflectometry derived polymer volume fraction profiles of a 210 Å PNIPAM brush in (a–c) 0.2 mol% electrolytes and d,e) 0.9 mol% electrolytes in a water‐methanol solvent composed of x_M_ = 0.10 at (a,d) 15.0 °C, (b,e) 25.0 °C, and (c,f) 32.5 °C. The corresponding scattering length density (SLD) profiles as well as the spread of potential fits, as derived from parallel‐tempered Markov chain Monte Carlo (PT‐MCMC) simulations, are presented in Figures S5.1‐S5.2 (Supporting Information).

In order to elucidate the impact of these electrolytes on polymer brush conformation, neutron reflectometry was performed to extract polymer volume fraction (VF) profiles of a 210 Å dry thickness PNIPAM brush. Resultant polymer VF profiles are presented in **Figure** **6**. The structure of the brush was probed in salt‐free *x*_M_ = 0.10, as well as KSCN, KCl, LiI, and LiBr *x*_M_ = 0.10 electrolytes at two different salt concentrations: a–c) 0.2 mol% and d–f) 0.9 mol%. Three temperatures were chosen (informed by Figure 3) to capture brush structure in the swollen, transition, and collapsed states: 15.0, 25.0, and 32.5 °C. Briefly, at 15 °C and in the absence of ions (i.e., *x*_M_ = 0.10; dark blue curve), the brush is swollen with a diffuse periphery. However, upon increasing the temperature to 25 and 32.5 °C, the periphery of the brush is seen to collapse, and the inner region increase; this yields a “slab‐like” structure, in line with the results presented in Figure 3.

As expected, exposing the brush to various electrolytes impacts brush conformation. The influence of electrolytes on brush structure is most distinct at lower temperatures and higher electrolyte concentrations, but the effects persist in the remaining conditions. Considering 15 °C and 0.9 mol% (i.e., Figure 6d), the brush in the presence of either KSCN or LiI is seen to be more swollen than in their absence: the periphery of the brush is more extended and the proximal/inner layers are less pronounced. In particular, KSCN is seen to impart a stronger salting‐in effect relative to LiI. This remains clear at 25 °C, with a persistent diffuse periphery; a mild salting‐in effect can also be seen at 32.5 °C. Conversely, upon exposing the brush to either KCl or LiBr a salting‐out effect is observed. At 0.9 mol% and 15 °C, both KCl and LiBr collapse the brush relative to their absence, increasing the proximal layer. Interestingly, a clear inner region is present when the brush is exposed to 0.9 mol% KCl whilst the inner region is suppressed when exposed to 0.9 mol% LiBr. Upon increasing the temperature to 25 and 32.5 °C, the salting‐out behavior persists with KCl imparting the strongest salting‐out effect. The brush is seen to be fully collapsed at 25 °C when exposed to 0.9 mol% KCl. These results concur with those deduced by spectroscopic ellipsometry (Figure 5), illustrating a *forward* Hofmeister series in *x*_M_ = 0.10 solvent. However, the forward Hofmeister series seen here for electrolytes in *x*_M_ = 0.10 was not observed by Mazzini and Craig, who show a reverse Hofmeister series in *x*_M_ = 1.0 via size exclusion chromatography in the absence of polymer.^[^ ^21^ ^]^ This is attributed to the presence of solvent clusters. Moreover, in contrast to the nanostructure of the brush in the absence of salt which showed the VF of polymer within the proximal layer to be relatively invariant across all temperatures (Figure 3), the polymer VF within the proximal layer in the presence of electrolytes readily alters, especially for the salting‐in ions at higher concentrations (e.g., Figure 6d,e). The reduction of polymer volume fraction (VF) within the proximal layer is attributed to the enhanced solvation and swelling of the polymer induced by SCN^−^ and I^−^ ions. This effect arises from a decrease in the adsorption of truncated polymers/oligomers at the silica interface and suggests that SCN^−^ and I^−^ facilitate a “bottom‐up” swelling mechanism within the polymer brush, aligning with the observed “bottom‐up” collapse of the brush.

Across both SE and NR, the addition of salts was seen to have a strong influence on polymer swelling (Figure 4) and nanostructure (Figure 6). For these water‐methanol mixtures, we build on our previous hypothesis regarding water‐DMSO electrolytes^[^ ^22^ ^]^ and conclude that salt addition to water‐methanol mixtures primarily affects the stability of solvent clusters, which, in turn, indirectly alters polymer conformation. As noted in §2.1, at *x*_M_ = 0.10, all methanol molecules are entrapped in water‐methanol solvent aggregates, meaning free solvent molecules are predominantly water molecules.

The structure and stability of these solvent aggregates are strongly influenced by the addition of ions. In particular, the addition of a strongly solvated ion with a high charge density, such as Br^−^, acts to both enhance the stability of water‐methanol clusters as well as further desolvate the polymer in favor of its solvation. This results in a net salting‐out effect on PNIPAM. Analogous behavior is observed for the addition of Cl^−^, however, its higher charge density (i.e., þ value) leads to a stronger salting‐out effect. Conversely, the addition of a poorly solvated ion with lower charge density, such as SCN^−^, acts to destabilize water‐methanol aggregates and stabilize PNIPAM. This yields a net increase in polymer swelling at a given temperature: salting‐in behavior. The addition of I^−^ to the system yields similar results. Whilst the addition of I^−^ does yield a more swollen brush profile (Figure 6) and impart a ∆LCST *> *0 (Figure 5), subtle decreases in brush thickness relative to the absence of I^−^ are observed at low temperatures in the ellipsometrically determined swelling profiles (Figure 4). The influence of all of these ions on the swelling and conformation of the PNIPAM is seen to increase with increasing concentration. These results also align with the previous investigations by Wang et al., who investigated the manifestation of SIE on untethered PNIPAM in water‐methanol mixtures.^[^ ^49^ ^]^ The SIE observed in *x*_M_ = 0.10 closely resemble those in pure water. However, while SIE in pure water are governed by ion‐polymer and ion‐water interactions,^[^ ^25^ ^]^ the SIE in *x*_M_ = 0.10 arise from the ions' ability to stabilize water‐methanol solvent clusters, which, in turn, enhance polymer stability.

Across all conditions examined the impact of the cation remains negligible relative to the anion. This is illustrated in Figure 5, which presents a linear relationship between ∆LCST and þ for each concentration series, despite the fact that Br^−^ and Cl^−^ have lithium counter‐cations, while SCN^−^ and I^−^ have potassium. If the cation imparted a stronger effect the data would deviate from linearity. This aligns with previous investigations of untethered PNIPAM in water‐methanol mixtures^[^ ^49^ ^]^ as well as a PNIPAM brush in water‐DMSO mixtures,^[^ ^22^ ^]^ where both K^+^ and Li^+^ were proposed to have a similar propensity to promote the formation of solvent‐water clusters.

**Figure 7:**
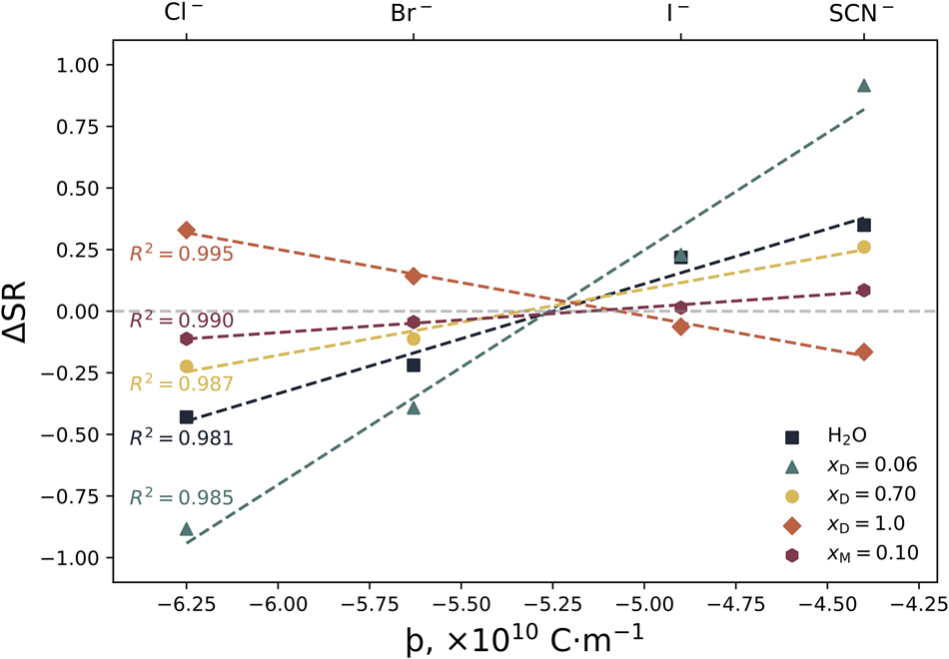
Change in the swelling ratio (∆SR) of a PNIPAM brush as a function of 0.2 mol% electrolyte identities composed of KCl, LiBr, LiI, and KSCN in solvents of water, 10 mol% MeOH (*x*_M_ = 0.10), 6 mol% DMSO (*x*_D_ = 0.06), 70 mol% DMSO (*x*_D_ = 0.70), and pure DMSO (*x*_D_ = 1.0). Lower *x*‐axis presents the ion's þ value and the upper *x*‐axis illustrates the corresponding anion identity. The change in SR is relative to the “no salt” condition in each solvent at the CST: 32.5 °C. for water, 26 °C for *x*_M_ = 0.10, 26 °C for *x*_D_ = 0.06, 55 °C for *x*_D_ = 0.70. An intermediate temperature of 26 °C was selected for *x*_D_ = 1.0, which does not exhibit a thermotransition. Uncertainties are smaller than the markers. A 322 Å brush was used for the measurements in *x*_M_ = 0.10 and a 714 Å brush was used for all other measurements. Data pertaining to DMSO solvents is recast with permission from Robertson et al.^[^ ^22^ ^]^

Linearity between brush thickness and anion identity is also apparent in **Figure** **7**, which presents the change in swelling ratio (∆SR) as a function of anion identity across various solvents. The behavior of a PNIPAM brush in water‐methanol electrolytes (*x*_M_) is presented here and is complemented by data from our previous work on PNIPAM brushes in water‐DMSO electrolytes (*x*_D_). An electrolyte concentration of 0.2 mol% was chosen to ensure solubility across all examined conditions. Here, a positive gradient represents the typical “forward” Hofmeister series, whereas a negative gradient is indicative of a “reverse” Hofmeister series. Interpolation of these data reveals a theoretical ion with a þ value of ≈5.2 × 10*^−^* ^10^ C m^−1^ which would impart a *null* impact on PNIPAM brush thickness. This null þ value can also be seen when considering ∆LCST by interpolating the data in Figure 5. In physical terms, this theoretical ion would have *no impact* on the thermoresponse or swelling of the polymer brush, allowing for variations in ionic strength without altering the behavior of the polymer itself. In line with our previous studies in water‐DMSO electrolytes,^[^ ^22^ ^]^ we reiterate our hypothesis that the persistence of this þ value of 5.2 × 10*^−^* ^10^ C m^−1^ throughout various solvent compositions (including pure DMSO) must be dominated by polymer‐ion interactions, rather than solvent‐ion interactions.

## 3 Conclusion

The thermoresponse and nanostructure of a PNIPAM brush were monitored as a function of solvent composition, electrolyte identity, and electrolyte strength. Initially, the swelling behavior of the brush was characterized in various water‐methanol solvent compositions, from pure water to pure methanol. Spectroscopic ellipsometry revealed that across the entire solvent composition range there are two apparent regimes. In water‐rich environments (*x*_M_ ≤ 0.15), PNIPAM exhibits LCST‐type behavior, where the LCST of the brush decreases with increasing *x*_M_. However, at high *x*_M_, the thermoresponse of the brush is suppressed and re‐entrant swelling is observed with increasing *x*_M_. Neutron reflectometry unveiled the subtle changes in brush nanostructure in *x*_M_ = 0.10 as a function of temperature.

The impact of electrolytes on brush swelling and conformation was then examined in a solvent composition of *x*_M_ = 0.10 with both spectroscopic ellipsometry and neutron reflectometry. In these water‐methanol solvents, the manifested specific ion effects were analogous to those observed in pure water: Cl^−^ and Br^−^ yielded a more collapsed brush, decreasing the LCST, whereas SCN^−^ and I^−^ yielded a more swollen brush, increasing the LCST. However, the manifestation of SIE primarily depended on the stability of solvent aggregates: Cl^−^ and Br^−^ stabilize the solvent aggregates, desolvating the polymer whereas SCN^−^ and I^−^ destabilize the solvent aggregates to stabilize the polymer. Interestingly, a theoretical electrolyte with a þ value of ≈5.2 × 10*^−^* ^10^ C m^−1^ would appear to impart a *null* effect on the thermotranstion and swelling of PNIPAM brush. This þ value transcends various electrolyte identities and solvent compositions.

## 4 Experimental Section

### Materials

Silicon blocks with an ≈1 nm native oxide layer (100 mm diameter; 10 mm thick) for neutron reflectometry were obtained from El‐Cat Inc. (USA). Silicon wafers with an ≈2 nm native oxide layer (0.675 mm thick) for spectroscopic ellipsometry were acquired from Silicon Valley Microelectronics (USA). Sodium hydroxide (NaOH) and ethanol were used for surface cleaning and purchased from Chem‐ Supply and Thermofisher Scientific, respectively. Surface functionalization reagents 2‐bromoisobutanoate bromide (BIBB, >99%), triethylamine (TEA, 99%), and (3‐aminopropyl)triethoxysilane (APTES, >99%) were procured from Sigma–Aldrich and used without further purification. Tetrahydrofuran (THF, >99%) was obtained from RCI Labscan Ltd and subjected to drying over 4 Å molecular sieves before use. Methanol was employed as a polymerization cosolvent and was sourced from Thermofisher Scientific, and used as received. Polymerization reagents (+)‐sodium *L*‐ascorbate (*>* 98%), copper bromide (CuBr_2_, 99.999%), and 1,1,4,7,10,10‐hexamethyltriethylenetetramine (HMTETA, 97%) were procured from Sigma–Aldrich and used as received. *N*‐isopropylacrylamide (NIPAM) monomer was acquired from Sigma–Aldrich and recrystallized from hexane (Sigma–Aldrich) prior to use. Potassium thiocyanate (KSCN, 99%), lithium iodide (LiI, 99.9%), and lithium bromide (LiBr, 99%) were obtained from Sigma–Aldrich whereas potassium chloride (KCl, 99%) was purchased from BDH Laboratory Supplies. All salts were pre‐dried at 110 °C prior to use. Methanol (MeOH, anhydrous, 99.9%) and deuterated methanol (d‐MeOH, anhydrous, 99.9 atom % D) were sourced from Sigma–Aldrich and used as received. MilliQ water (Merck Millipore, 18.2 MΩ cm at 25 °C) served as the solvent throughout, except for neutron reflectometry experiments which used D_2_O (Sigma‐Aldrich). All glassware was thoroughly washed with MilliQ water and ethanol, followed by immersion in a 10% HNO_3_ acid bath for a minimum of 24 h before use.

### Polymer Brush Synthesis

All polymer brushes for spectroscopic ellipsometry and neutron reflectometry were synthesized according to the previously reported method.^[^ ^22^, ^24^, ^38^, ^50^ ^]^ To achieve appropriate polymerization kinetics, the methanol/water solvent ratio was chosen as 4:1 v/v and the monomer/catalyst/ligand/reducing agent molar ratio was 900/1/10/10 with NIPAM/CuBr_2_/HMTETA/sodium ascorbate. Table 1 presents a summary of the dry film thickness (measured at standard laboratory conditions: *T* = 20 °C; *RH* = 30%) for each PNIPAM brush. All samples are denoted by their dry brush thickness, quantified through air‐solid ellipsometry, and outlined in Table 1.

### Ellipsometry

Ellipsometry measurements were conducted using the Accurion EP4 variable angle spectroscopic imaging ellipsometer located at the Australian Center for Neutron Scattering (ANSTO, Lucas Heights, Australia).^[^ ^22^, ^24^, ^38^, ^51^ ^]^ Surface mapping of dry films for all brushes was executed at a consistent wavelength (658 nm) over 31 surface points, employing four evenly spaced angles of incidence ranging from 40° to 70°. Spectroscopic in situ measurements focused on a single polymer brush within the standard solid‐liquid cell. The incident radiation was set at 65° perpendicular to the surface and employed ten equally spaced wavelengths ranging from 400 to 900 nm. Temperature ramps were systematically increased, and the experimental conditions followed a sequence of increasing MeOH content. A salt‐free solvent flush was conducted during transitions to new salts. The choice of salts and their concentrations studied reflect their solubility in a broader spectrum of solvents in a greater set of studies.^[^ ^22^ ^]^

Ellipsometry data were analyzed with the *refellips* software package.^[^ ^52^ ^]^ In short, all data were modeled using four uniform layers (slabs), which describe the optical properties, the volume fraction of solvent present (if applicable), as well as thickness and roughness. For measurements in air, the model structure was air, polymer, silica, silicon from “fronting” to “backing” (i.e., in the direction of irradiation).^[^ ^22^ ^]^ For in situ solid‐liquid measurements, the slab representing the fronting medium was simply modified to the respective solvent present. For both dry and in situ measurements, the model optical properties of the polymer brush were set using a Cauchy model, with parameters fixed at *A* = 1.45*, B* = 0.005. The optical properties of the solvent were also described using a Cauchy model, where parameters were permitted to vary to allow for slight deviations in refractive index as methanol was incorporated.

When exposed to solvents composed of water and water‐rich methanol mixtures, PNIPAM exhibits a lower critical solution temperature (LCST) thermoresponse.^[^ ^6^, ^35^, ^36^ ^]^ The LCST of PNIPAM in various electrolytes with a solvent composition of *x*_M_ = 0.1 was extracted as *g* from a logistic function:

(1) $$y\left(t\right)=a+\frac{b-a}{c+d\;\exp{\left(-e\left(t-g\right)\right)}^{1/f}}$$

A detailed explanation of this analysis can be found in previous works.^[^ ^22^, ^38^ ^]^

### Neutron Reflectometry

Specular neutron reflectometry (NR) data were gathered using the *PLATYPUS* time‐of‐flight neutron reflectometer at the 20 MW OPAL nuclear reactor, Australian Centre for Neutron Scattering, ANSTO, Lucas Heights, Australia.^[^ ^53^ ^]^ Air‐solid measurements were collected at two angles of incidence, 0.65° and 3.0°, which were stitched together to cover a *Q*‐range from 0.008 to 0.25 Å*^−^* ^1^. In situ measurements were also collected at two angles of incidence, 0.8° and 3.2°, which spanned a *Q*‐range from 0.01 to 0.30 Å. All measurements had a fixed d*θ*/*θ* of 0.033 and a footprint of 65 mm. Following the established methodology,^[^ ^24^, ^38^, ^47^, ^51^ ^]^ samples were housed in standard solid‐liquid cells with temperature jackets, and reflectivity was measured in an upward‐reflecting geometry. Temperature ramps were conducted in line with spectroscopic ellipsometry data, and each electrolyte addition was preceded by flushing the cell with pure solvent.

Temporal acquisitions were collected using event mode reduction in alignment with the established protocol.^[^ ^46^ ^]^ These measurements aid in surveying the collapse of the polymer brush over a wide temperature range, ultimately informing conditions for subsequent measurements. The temperature was varied from 13 to 40 °C at 0.5 °C min*^−^* ^1^ while acquiring NR data (PLATYPUS reflectometer, *θ* = 0.8°, *Q* = 0.0017 Å*^−^* ^1^ to 0.078 Å^−1^). Data were reduced into 2 min time slices.

### Neutron Reflectometry Analysis

All neutron reflectometry data were reduced and modeled with the *refnx* software package^[^ ^54^ ^]^ and informed by the previous investigations.^[^ ^22^, ^38^, ^47^, ^51^ ^]^ Briefly, components were used to describe each layer within the system, describing the respective thickness, roughness, volume fraction (VF) of solvent, and scattering length density (SLD) of each layer. For dry measurements, the models used were consistent with those employed for spectroscopic ellipsometry, using only slab components; from “fronting” to “backing”, the structure was air, polymer, silica, and air. For in situ measurements, a piecewise cubic Hermite interpolating polynomial (PCHIP) was employed to describe the diffuse periphery of the brush in conjunction with slabs.^[^ ^47^ ^]^ For these in situ measurements, the structure was reversed, with the air slab being replaced with a solvent slab. The diffuse brush periphery is described by a PCHIP spline.^[^ ^47^ ^]^ Consistent with previous investigations,^[^ ^47^, ^51^, ^55^ ^]^ the PCHIP spline contained four knots (spline control point), whereby the volume fraction and distance between each knot were permitted to vary. Monotonicity was enforced across all conditions, and the dry brush thickness constrained the adsorbed interfacial volume (*δ*_dry_) (Table 1). The brush thickness was then extracted from these polymer VF profiles as twice the first moment, which was calculated via:

(2) $$L_{1\mathrm{st}}=2\frac{\underset{0}{\int^{\infty }}z\cdot \phi \left(z\right)\mathrm{dz}}{\delta_{\mathrm{dry}}}$$

where *z* is the orthogonal distance from the substrate and *z* = 0 represents the interface between the oxide and polymer.

From the polymer VF profile, the theoretical SLD profile (*ρ*_N_(*z*)) could be calculated by:^[^ ^47^ ^]^

(3) $$\rho_{N}\left(z\right)=\phi \left(z\right)\rho_{N,\mathrm{Polymer}}+\left(1-\phi \left(z\right)\right)\rho_{N,\mathrm{Solvent}}$$

where *ρ*_N_ *_,_*_Solvent_ and *ρ*_N_ *_,_*_Polymer_ represent the neutron SLD of the solvent and polymer, respectively. From the calculated SLD profile, the Abeles matrix formalism was employed to determine the reflectivity profile. This theoretical reflectivity profile was then optimized against the experimental reflectivity via differential evolution. Subsequent parallel tempered Markov chain Monte Carlo (PT‐MCMC) simulations were then employed to sample the posterior distribution of the data, furnishing the distribution of structures consistent with the data. All optimized models and polymer VF profiles presented in this study are extracted as the median of the PT‐MCMC samples, which all showed narrow distributions. These are presented in Figures S5.1–S5.2 (Supporting Information). A more detailed explanation of this approach is explored by Gresham et al.,^[^ ^47^ ^]^ and all relevant data and code required to reproduce these analyses are available on the Zenodo repository.^[^ ^56^ ^]^

## Conflict of Interest

The authors declare no conflict of interest.

## Supporting information



Supporting Information

## Data Availability

The data that support the findings of this study are openly available in [Zenodo] at [https://doi.org/10.5281/zenodo.14338739], reference number [14338739].
