# Supplementary material for: Solvent and Ion‐Mediated Behavior of a Thermoresponsive Brush: Specific Ion Effects in Methanol‐Water Electrolytes
Source: Macromol Rapid Commun. 2025 May 13;46(16):2500093. doi: 10.1002/marc.202500093 (PMC12360131; doi:10.1002/marc.202500093)
Supplement: Supplementary file 1 — Supporting Information [file MARC-46-2500093-s001.pdf]

Macromolecular Rapid Communications

Supporting Information

for *Macromol. Rapid Commun.*, DOI 10.1002/marc.202500093

Solvent and Ion-Mediated Behavior of a Thermoresponsive Brush: Specific Ion Effects in Methanol-Water Electrolytes

*Hayden Robertson, Joshua D. Willott, Andrew R.J. Nelson, Stuart W. Prescott, Erica J. Wanless and Grant, B. Webber**

SI for "Solvent and ion-mediated behavior of a thermoresponsive brush: Specific ion effects in methanol-water electrolytes"

Hayden Robertson[a,b], Joshua D. Willott[b], Andrew R. J. Nelson[c], Stuart W. Prescott[d], Erica J. Wanless[b], and Grant B. Webber[b,*]

[a] *Soft Matter at Interfaces, Technical University of Darmstadt, Darmstadt D-64289, Germany*
[b] *College of Science, Engineering and Environment, University of Newcastle, Callaghan, Australia*
[c] *Australian Centre for Neutron Scattering, ANSTO, Locked Bag 2001, Kirrawee DC, NSW 2232, Australia*
[d] *School of Chemical Engineering, UNSW Sydney, NSW 2052, Australia*
[*] *email: grant.webber@newcastle.edu.au*

All relevant data and code required to reproduce the analyses presented are readily available on Zenodo at `https://doi.org/10.5281/zenodo.14338739`.

Contents

1 þ values **2**

2 Fourier-transform infrared spectroscopy **2**

3 Ellipsometry **3**

4 Additional NR polymer VF profiles **4**

5 NR PT-MCMC Spread of fits **6**

5.1 D_2O ($x_M = 0$) 6

5.2 10 mol% methanol ($x_M = 0.10$) 7

References **16**

1 þ values

Table S1.1: Relevant þ values for this work. All values are taken from the work of Gregory *et al.*[1]

Ion identity	þ, C·m^{-1}
Cl^-	-6.25×10^{-10}
Br^-	-5.63×10^{-10}
I^-	-4.90×10^{-10}
SCN^-	-4.40×10^{-10}
K^+	9.24×10^{-10}
Li^+	31.61×10^{-10}

2 Fourier-transform infrared spectroscopy

Fourier-transform infrared (FTIR) spectroscopy was carried out using a Perkin-Elmer FTIR system. Measurements were repeated five times for each sample, covering the spectral range of 400 cm^{-1} to 4000 cm^{-1} with a resolution of 1 cm^{-1}. This technique was applied to analyse water-methanol mixtures across all composition ratios. Figure S2.1 displays the full spectrum of each sample with the inset highlighting region from 980 cm^{-1} to 1080 cm^{-1}.

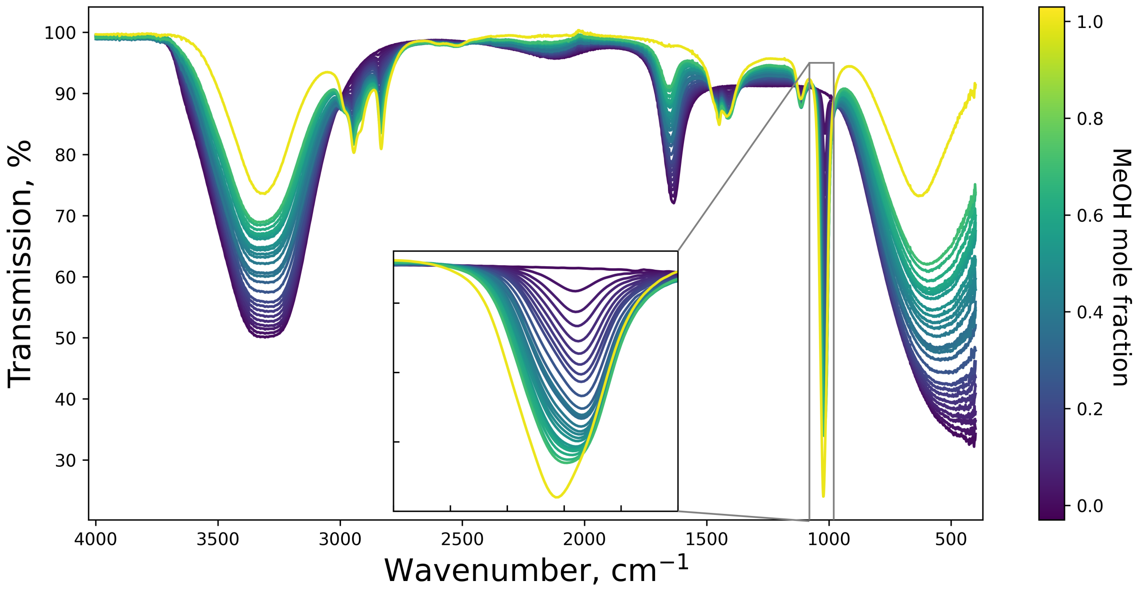

Figure S2.1: FTIR spectra of various water-methanol mixtures. The magnified inset presents the C–O stretch from 980 cm^{-1} to 1080 cm^{-1}.

3 Ellipsometry

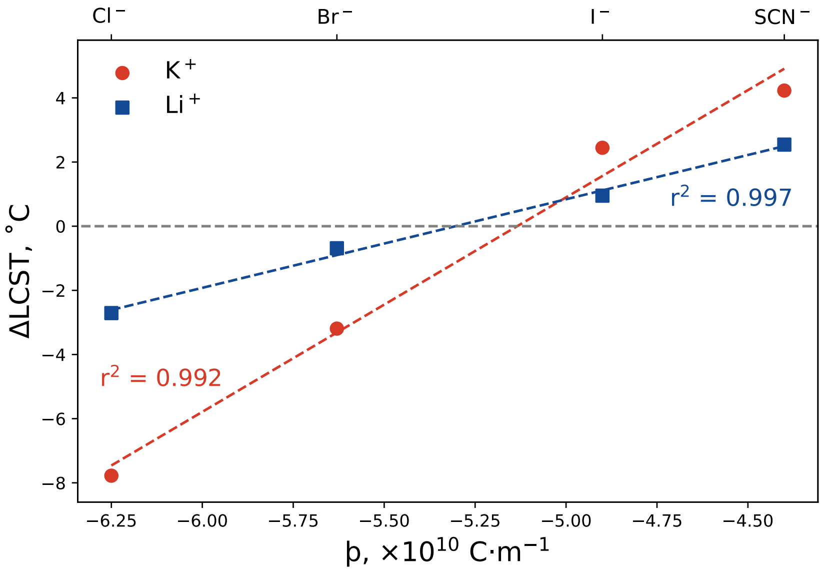

Figure S3.1: Change in LCST of a 306 Å PNIPAM brush in various 0.9 mol% aqueous electrolytes. Top x-axis identifies ions probed and lower x-axis shows their respective þ value. Dashed lines are linear fits for potassium (K^+; red circles) and lithium (Li^+; blue squares) data. Figure reproduced with permission from Robertson *et al.*.[2]

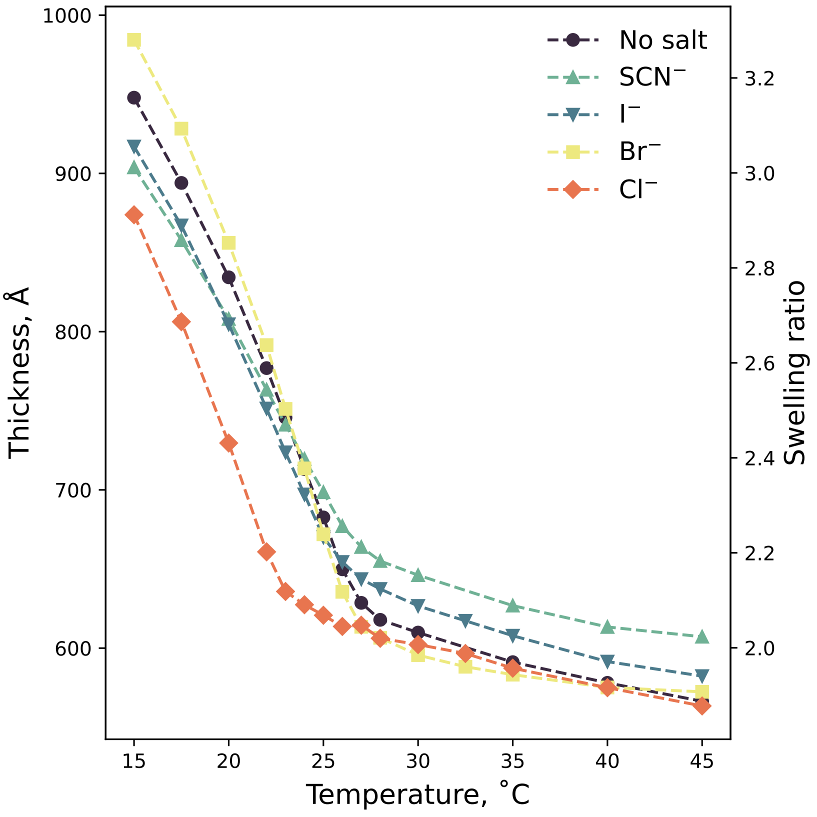

Figure S3.2: Ellipsometrically determined brush thickness (left) and swelling ratio (right) of a 322 Å PNIPAM brush exposed to 0.2 mol% KSCN, LiI, LiBr, and KCl electrolytes in water-methanol composed of $x_M = 0.10$ as a function of temperature.

4 Additional NR polymer VF profiles

All neutron reflectometry was modelled using *refnx* in concordance with our previous protocols.[3,4] Key conditions were examined with event-mode acquisition and analysed with correlation analysis according to our previously established protocol: data was acquired at an angle of incidence of 0.8° with 2% resolution whilst the temperature was increased from approximately 15 °C to at least 22 °C, binned at 2 min intervals.[5] Clear changes in the reflectivity of the brush during collapse as seen in Figure S4.1 (i.e., swollen and featureless to collapsed with Kiessig fringes) permit this analysis. Here the 'similarity index' is defined as the normalised cross-correlation of reflectivity at zero lag relative to the most collapsed case. Further information on this analysis is discussed by Robertson et al.[5] Figure S4.2a presents the similarity index between reflectivity profiles of a PNIPAM brush in various solvents; Figure S4.2b shows the extracted LCST values.

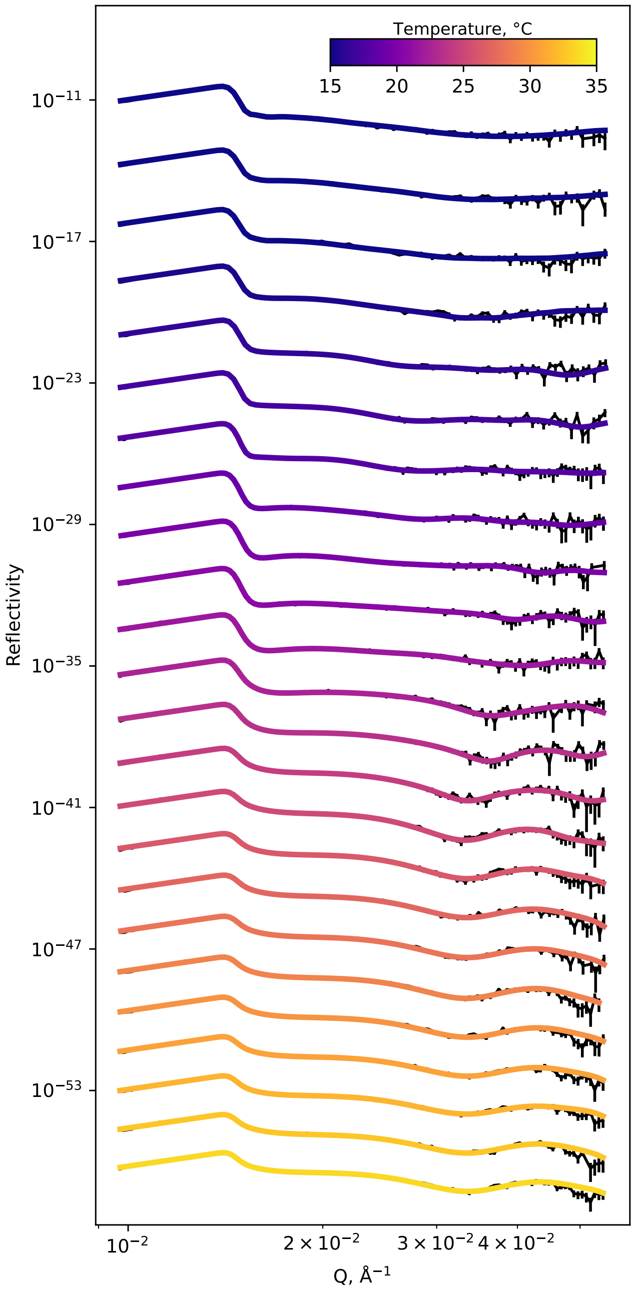

Figure S4.1: Corresponding experimental and modelled reflectivity of a 210 Å PNIPAM brush in $x_{\mathrm{M}} = 0.10$ for the polymer VF profiles presented in Figure 3.

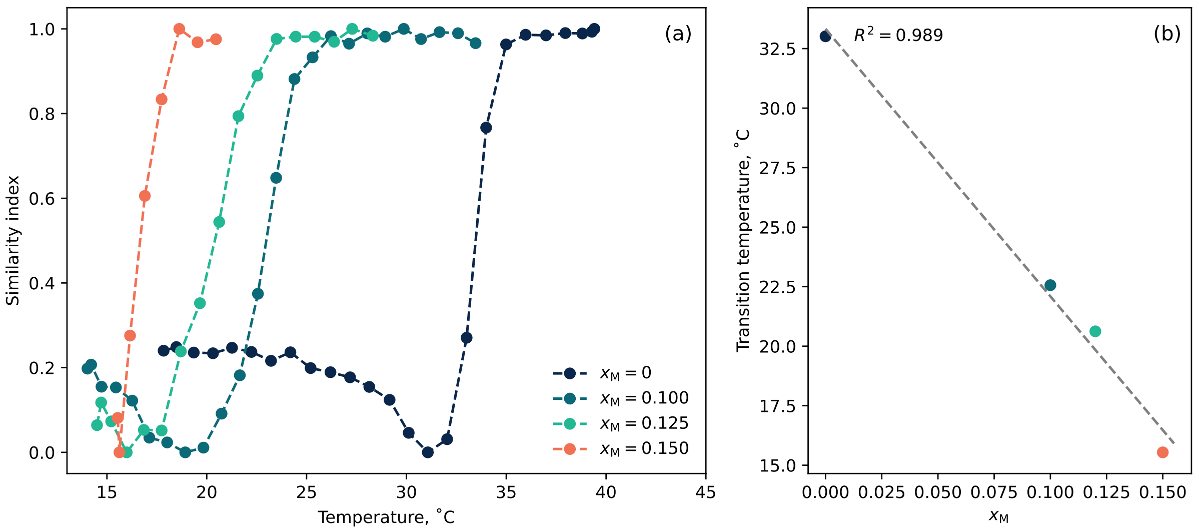

Figure S4.2: *In situ* correlation analysis of a 210 Å PNIPAM brush in solvents of various water-methanol mole fraction (x_M). (a) The similarity index as a function of temperature and x_M, and (b) the resultant transition temperature from the instantaneous gradient in (a) with the largest magnitude.

5 NR PT-MCMC Spread of fits

5.1 D_2O ($x_M = 0$)

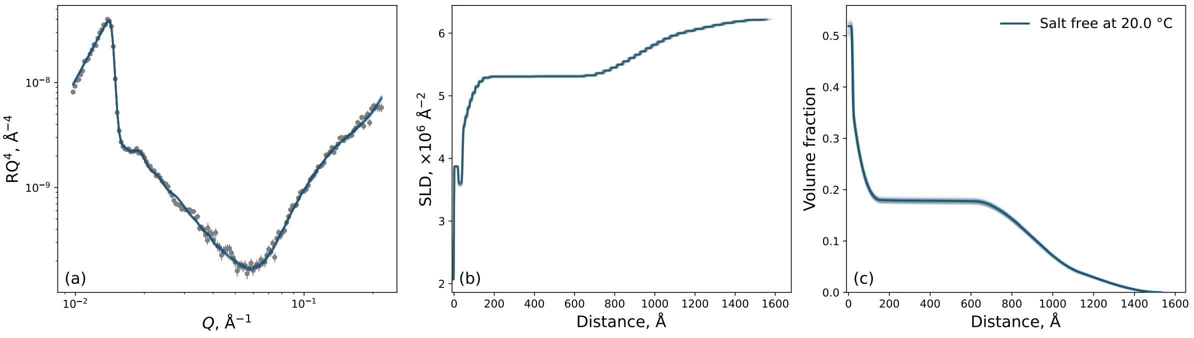

Figure S5.1i: (a) Reflectivity, (b) SLD and (c) polymer VF profiles of the 210 Å PNIPAM brush in D_2O at 20.0 °C with the superimposed distribution of fits from PT-MCMC sampling.

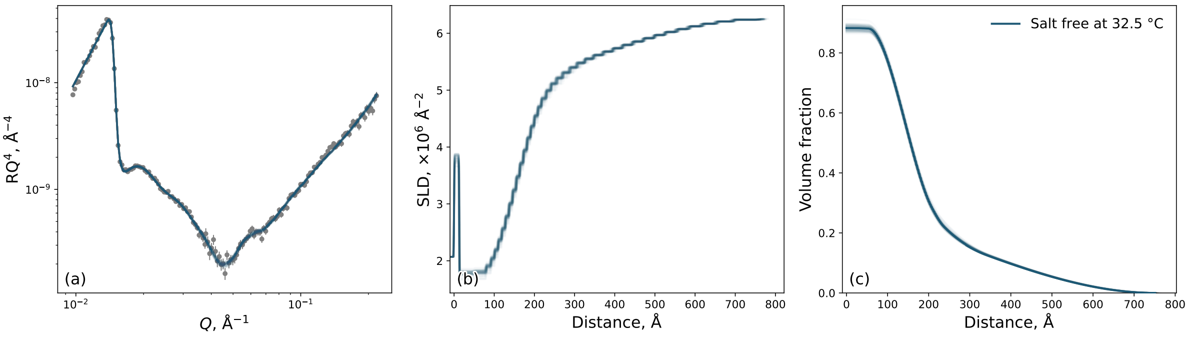

Figure S5.1ii: (a) Reflectivity, (b) SLD and (c) polymer VF profiles of the 210 Å PNIPAM brush in D_2O at 32.5 °C with the superimposed distribution of fits from PT-MCMC sampling.

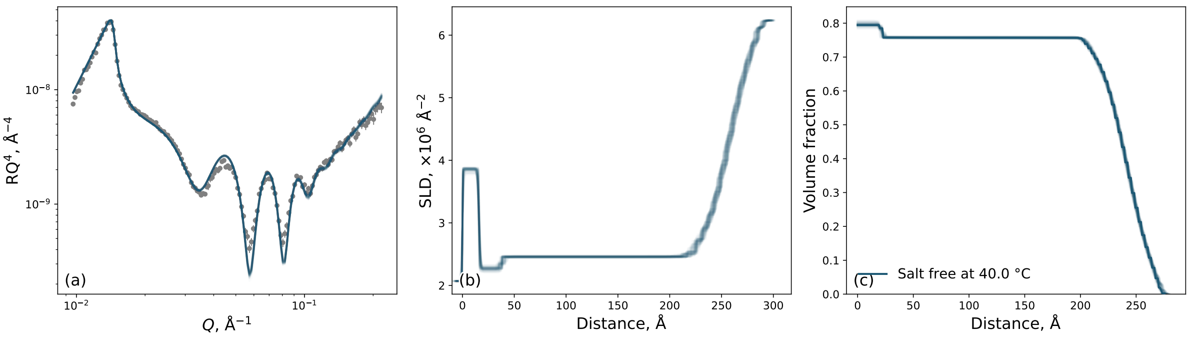

Figure S5.1iii: (a) Reflectivity, (b) SLD and (c) polymer VF profiles of the 210 Å PNIPAM brush in D_2O at 40.0 °C with the superimposed distribution of fits from PT-MCMC sampling.

5.2 10 mol% methanol ($x_M = 0.10$)

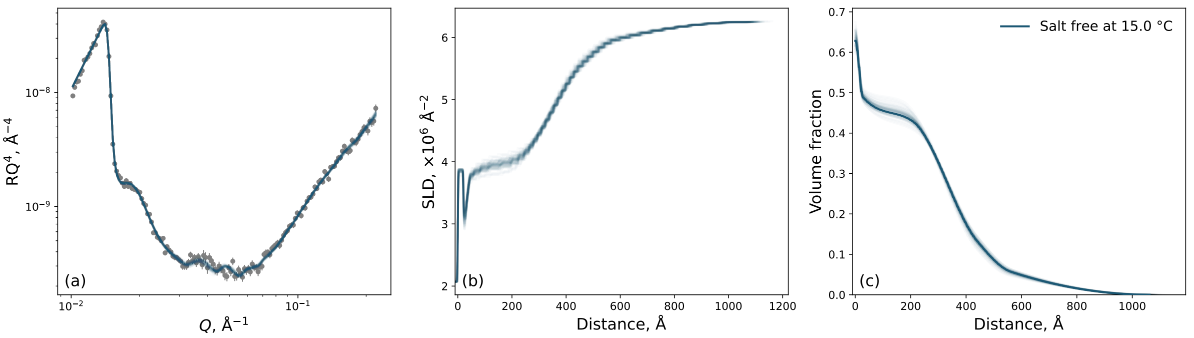

Figure S5.2i: (a) Reflectivity, (b) SLD and (c) polymer VF profiles of the 210 Å PNIPAM brush in $x_M = 0.10$ at 15.0 °C with the superimposed distribution of fits from PT-MCMC sampling.

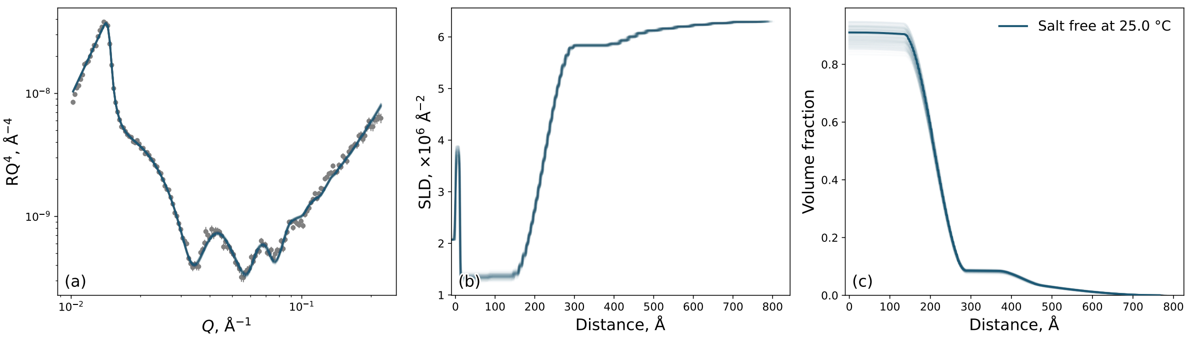

Figure S5.2ii: (a) Reflectivity, (b) SLD and (c) polymer VF profiles of the 210 Å PNIPAM brush in $x_M = 0.10$ at 25.0 °C with the superimposed distribution of fits from PT-MCMC sampling.

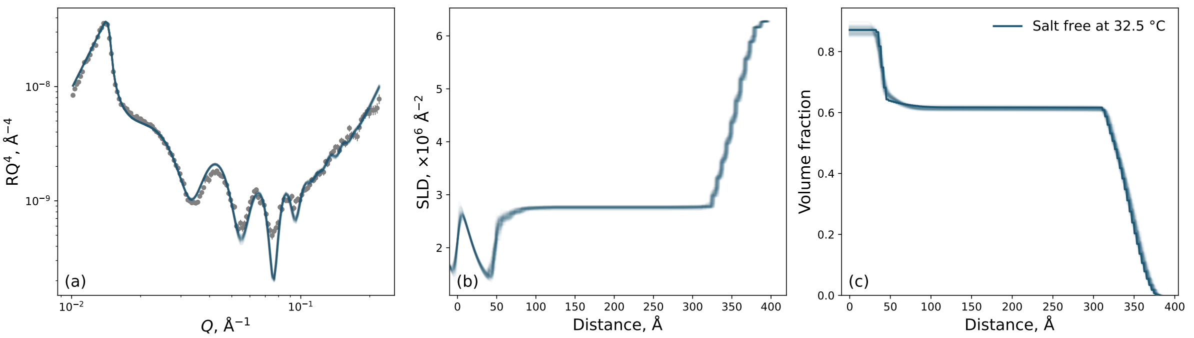

Figure S5.2iii: (a) Reflectivity, (b) SLD and (c) polymer VF profiles of the 210 Å PNIPAM brush in $x_M = 0.10$ at 32.5 °C with the superimposed distribution of fits from PT-MCMC sampling.

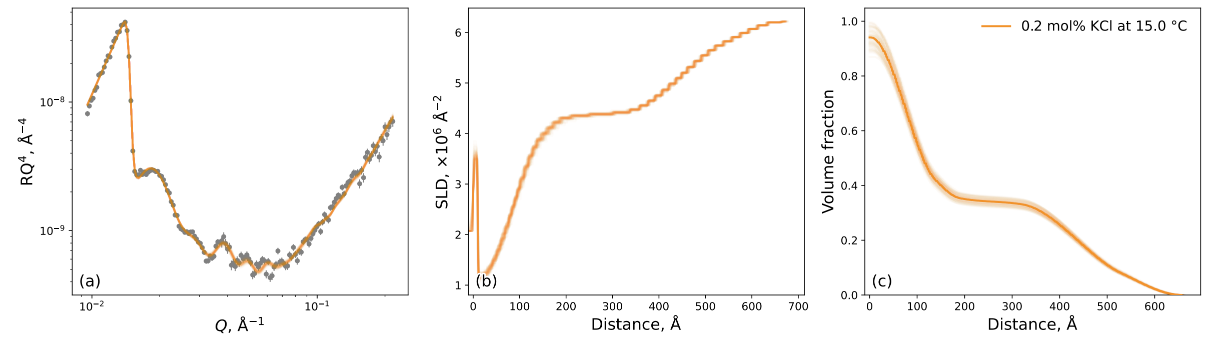

Figure S5.2iv: (a) Reflectivity, (b) SLD and (c) polymer VF profiles of the 210 Å PNIPAM brush in a 0.2 mol% KCl electrolyte in $x_{\mathrm{M}} = 0.10$ at 15.0 °C with the superimposed distribution of fits from PT-MCMC sampling.

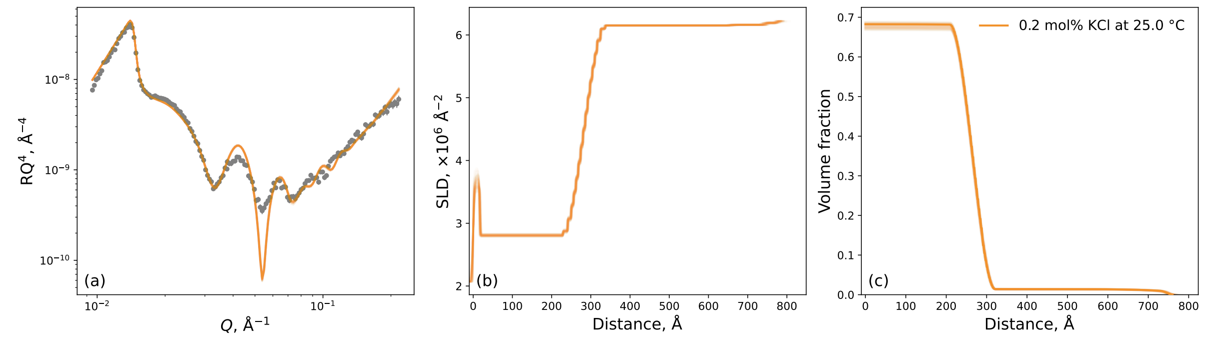

Figure S5.2v: (a) Reflectivity, (b) SLD and (c) polymer VF profiles of the 210 Å PNIPAM brush in a 0.2 mol% KCl electrolyte in $x_{\mathrm{M}} = 0.10$ at 25.0 °C with the superimposed distribution of fits from PT-MCMC sampling.

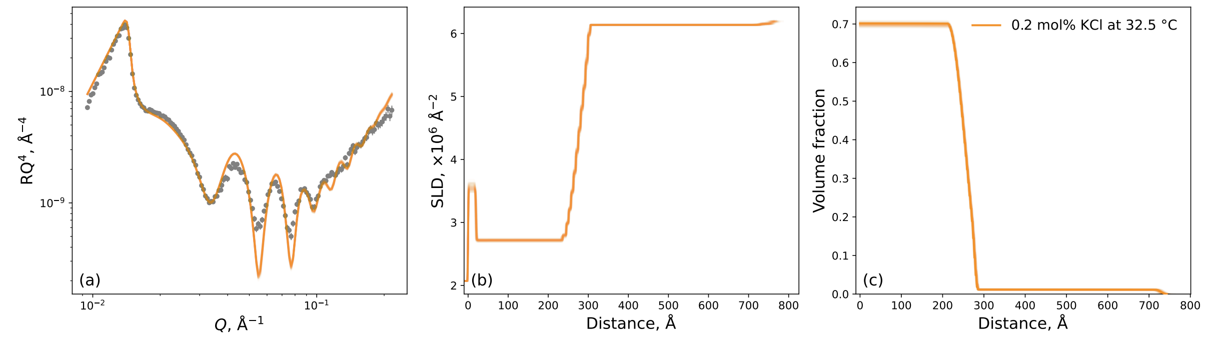

Figure S5.2vi: (a) Reflectivity, (b) SLD and (c) polymer VF profiles of the 210 Å PNIPAM brush in a 0.2 mol% KCl electrolyte in $x_{\mathrm{M}} = 0.10$ at 32.5 °C with the superimposed distribution of fits from PT-MCMC sampling.

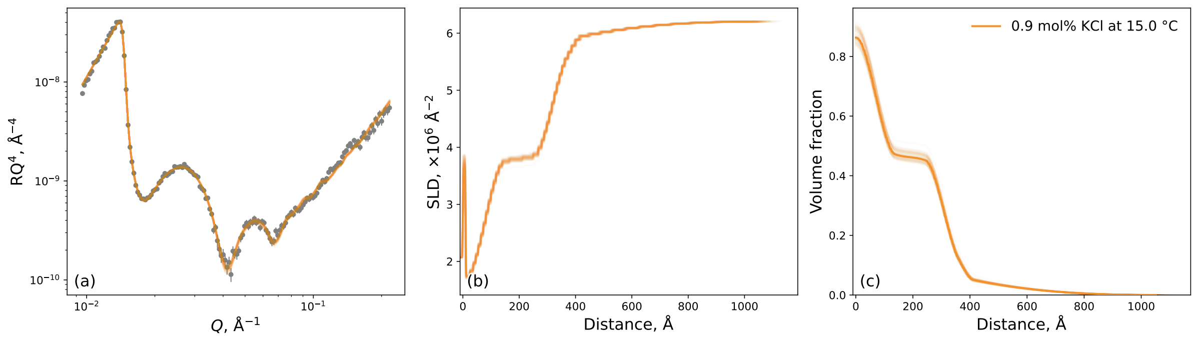

Figure S5.2vii: (a) Reflectivity, (b) SLD and (c) polymer VF profiles of the 210 Å PNIPAM brush in a 0.9 mol% KCl electrolyte in $x_M = 0.10$ at 15.0 °C with the superimposed distribution of fits from PT-MCMC sampling.

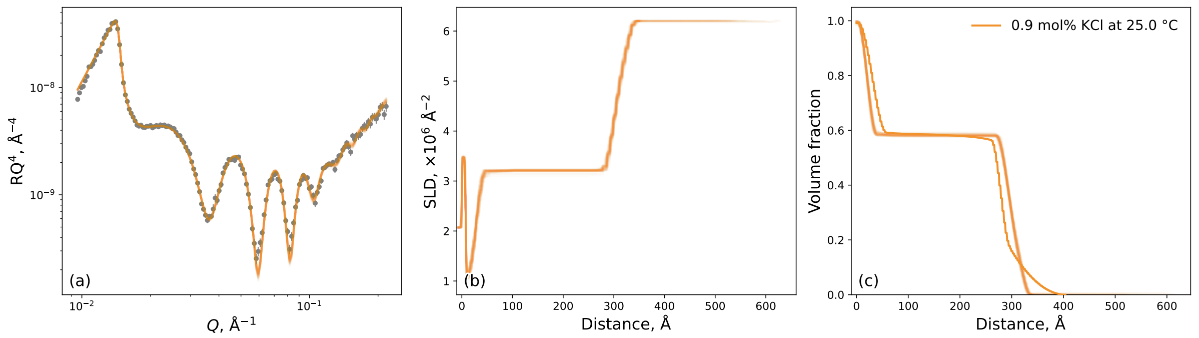

Figure S5.2viii: (a) Reflectivity, (b) SLD and (c) polymer VF profiles of the 210 Å PNIPAM brush in a 0.9 mol% KCl electrolyte in $x_M = 0.10$ at 25.0 °C with the superimposed distribution of fits from PT-MCMC sampling.

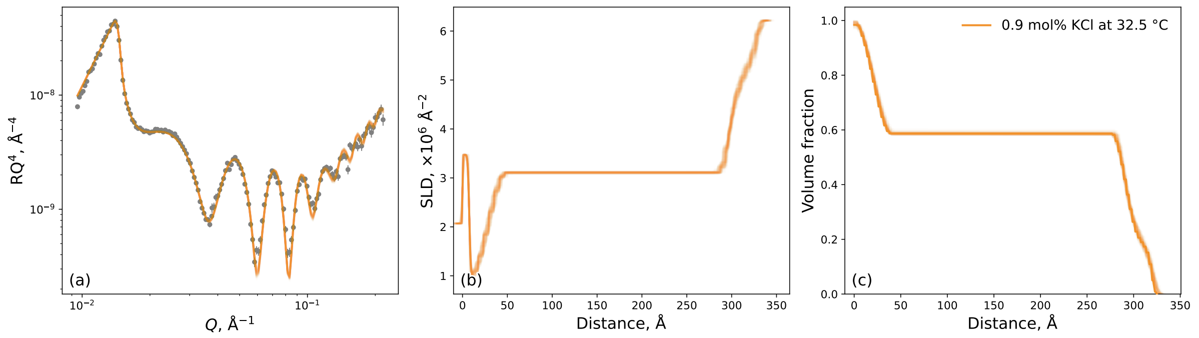

Figure S5.2ix: (a) Reflectivity, (b) SLD and (c) polymer VF profiles of the 210 Å PNIPAM brush in a 0.9 mol% KCl electrolyte in $x_M = 0.10$ at 32.5 °C with the superimposed distribution of fits from PT-MCMC sampling.

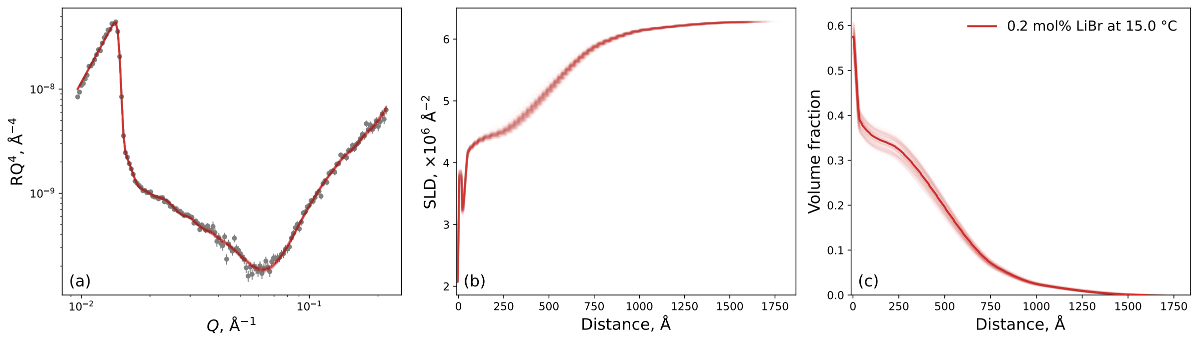

Figure S5.2x: (a) Reflectivity, (b) SLD and (c) polymer VF profiles of the 210 Å PNIPAM brush in a 0.2 mol% LiBr electrolyte in $x_{\mathrm{M}} = 0.10$ at 15.0 °C with the superimposed distribution of fits from PT-MCMC sampling.

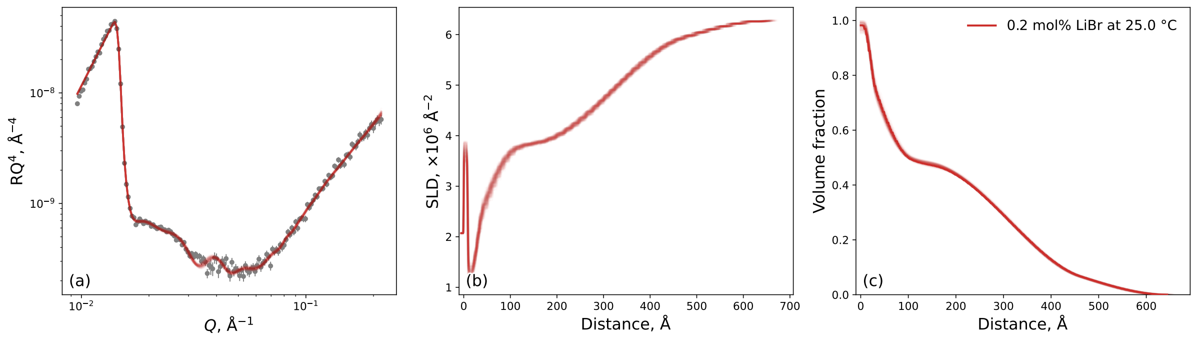

Figure S5.2xi: (a) Reflectivity, (b) SLD and (c) polymer VF profiles of the 210 Å PNIPAM brush in a 0.2 mol% LiBr electrolyte in $x_{\mathrm{M}} = 0.10$ at 25.0 °C with the superimposed distribution of fits from PT-MCMC sampling.

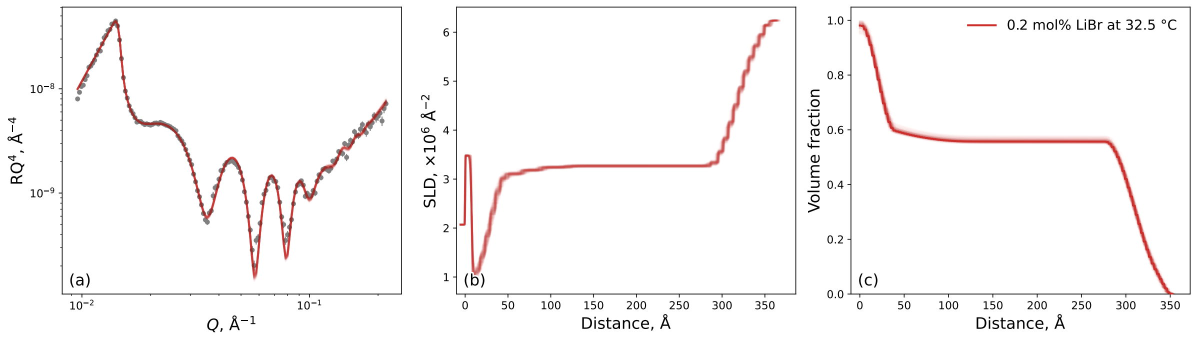

Figure S5.2xii: (a) Reflectivity, (b) SLD and (c) polymer VF profiles of the 210 Å PNIPAM brush in a 0.2 mol% LiBr electrolyte in $x_{\mathrm{M}} = 0.10$ at 32.5 °C with the superimposed distribution of fits from PT-MCMC sampling.

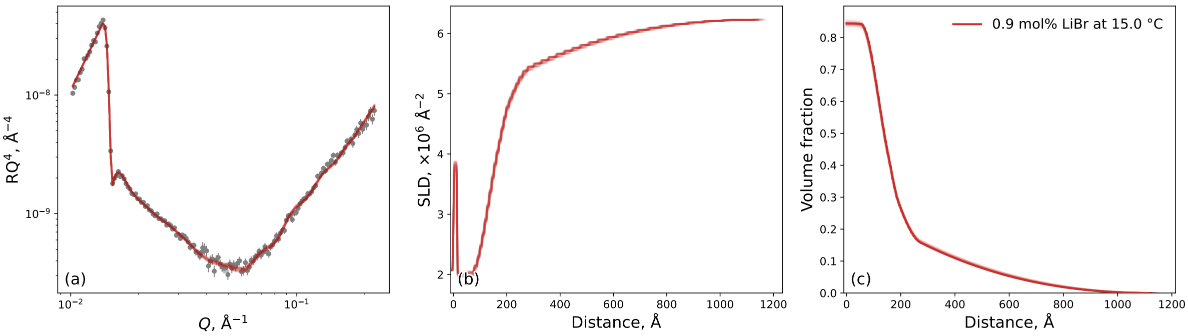

Figure S5.2xiii: (a) Reflectivity, (b) SLD and (c) polymer VF profiles of the 210 Å PNIPAM brush in a 0.9 mol% LiBr electrolyte in $x_{\mathrm{M}} = 0.10$ at 15.0 °C with the superimposed distribution of fits from PT-MCMC sampling.

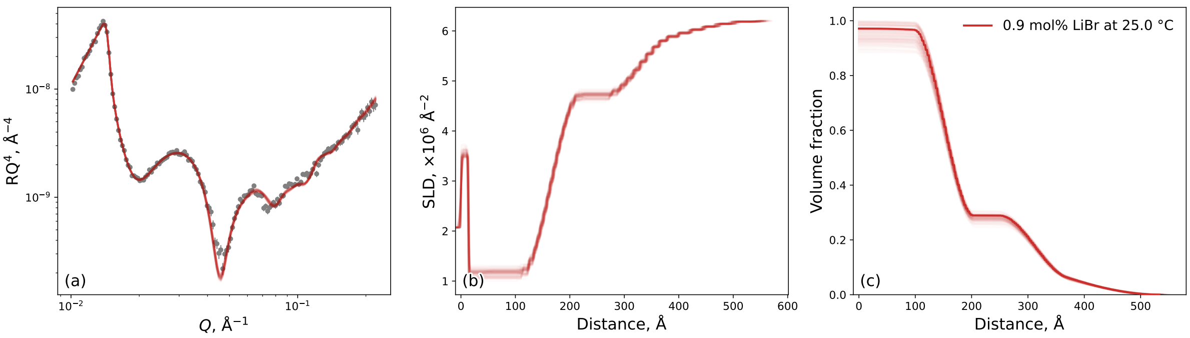

Figure S5.2xiv: (a) Reflectivity, (b) SLD and (c) polymer VF profiles of the 210 Å PNIPAM brush in a 0.9 mol% LiBr electrolyte in $x_{\mathrm{M}} = 0.10$ at 25.0 °C with the superimposed distribution of fits from PT-MCMC sampling.

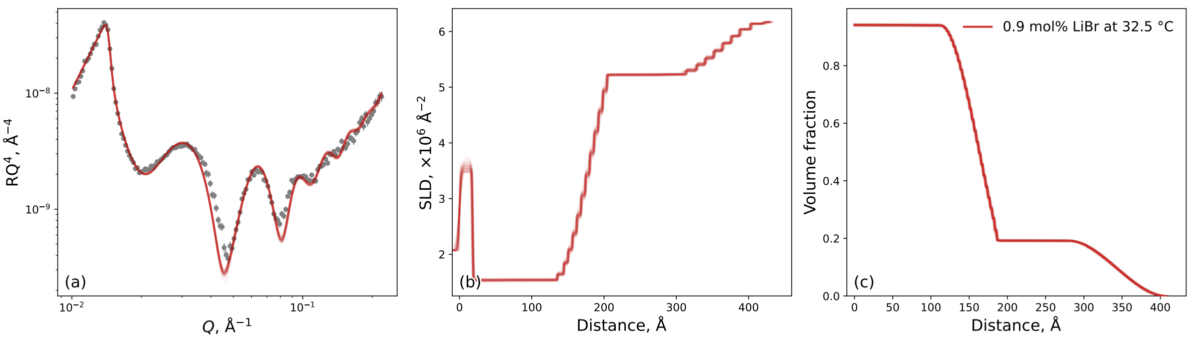

Figure S5.2xv: (a) Reflectivity, (b) SLD and (c) polymer VF profiles of the 210 Å PNIPAM brush in a 0.9 mol% LiBr electrolyte in $x_{\mathrm{M}} = 0.10$ at 32.5 °C with the superimposed distribution of fits from PT-MCMC sampling.

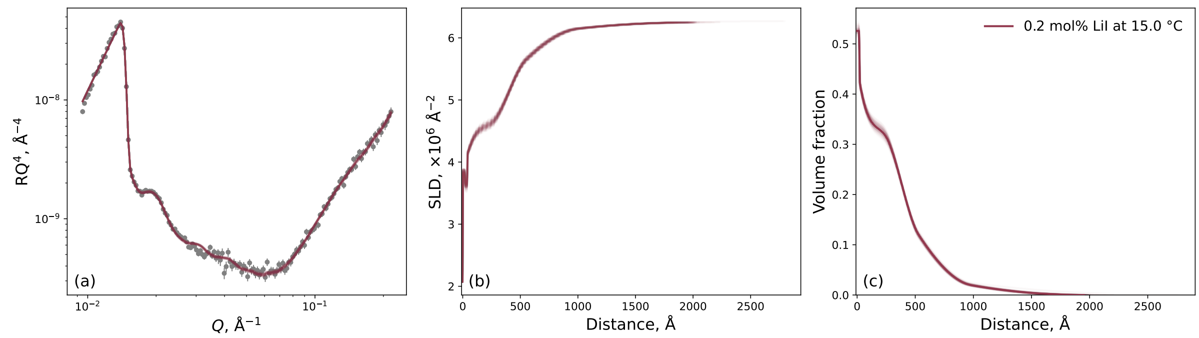

Figure S5.2xvi: (a) Reflectivity, (b) SLD and (c) polymer VF profiles of the 210 Å PNIPAM brush in a 0.2 mol% LiI electrolyte in $x_{\mathrm{M}} = 0.10$ at 15.0 °C with the superimposed distribution of fits from PT-MCMC sampling.

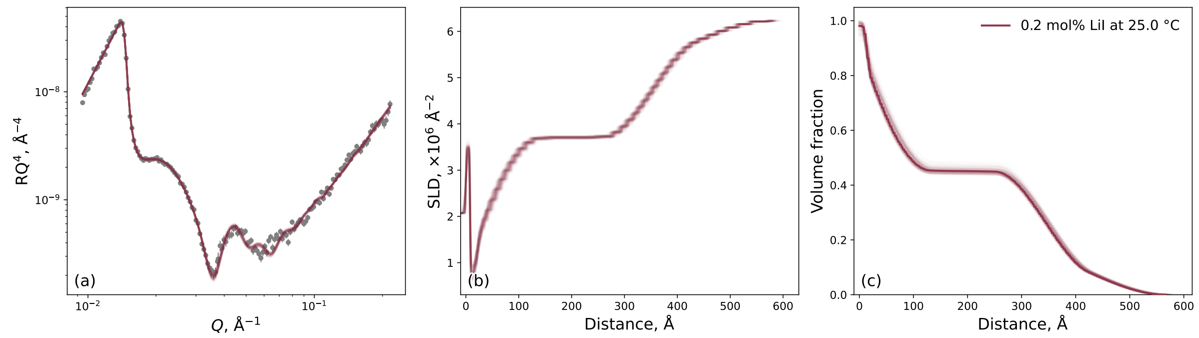

Figure S5.2xvii: (a) Reflectivity, (b) SLD and (c) polymer VF profiles of the 210 Å PNIPAM brush in a 0.2 mol% LiI electrolyte in $x_{\mathrm{M}} = 0.10$ at 25.0 °C with the superimposed distribution of fits from PT-MCMC sampling.

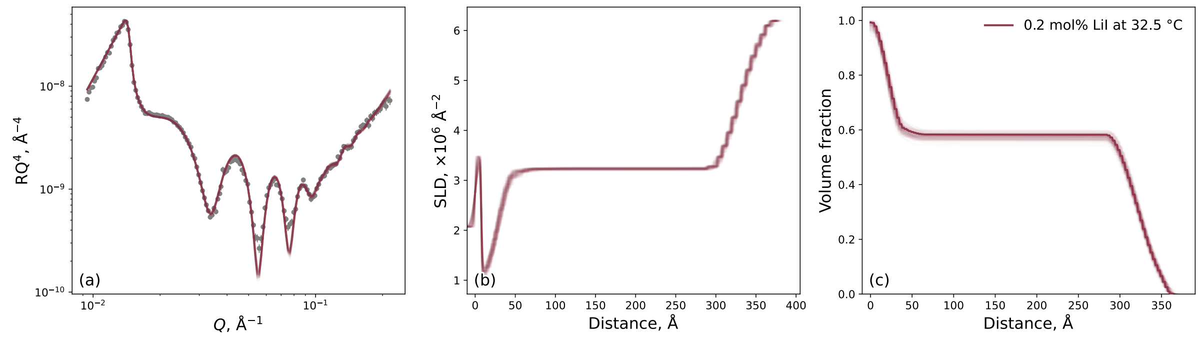

Figure S5.2xviii: (a) Reflectivity, (b) SLD and (c) polymer VF profiles of the 210 Å PNIPAM brush in a 0.2 mol% LiI electrolyte in $x_{\mathrm{M}} = 0.10$ at 32.5 °C with the superimposed distribution of fits from PT-MCMC sampling.

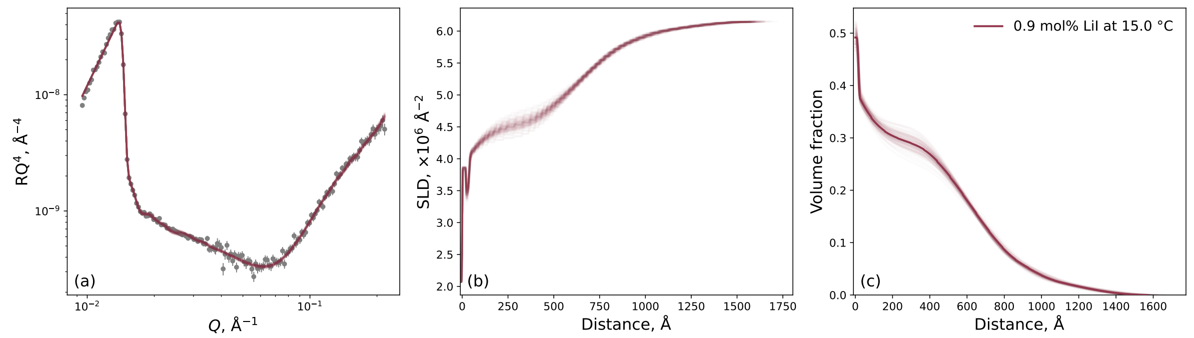

Figure S5.2xix: (a) Reflectivity, (b) SLD and (c) polymer VF profiles of the 210 Å PNIPAM brush in a 0.9 mol% LiI electrolyte in $x_\mathrm{M} = 0.10$ at 15.0 °C with the superimposed distribution of fits from PT-MCMC sampling.

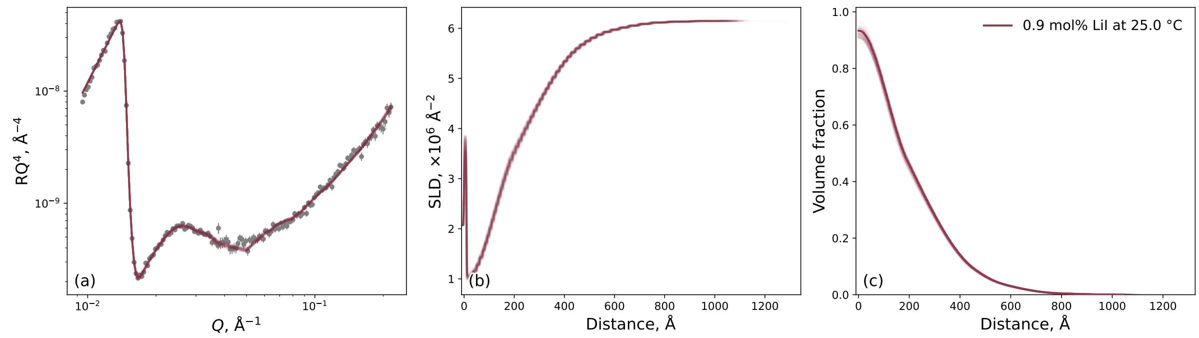

Figure S5.2xx: (a) Reflectivity, (b) SLD and (c) polymer VF profiles of the 210 Å PNIPAM brush in a 0.9 mol% LiI electrolyte in $x_\mathrm{M} = 0.10$ at 25.0 °C with the superimposed distribution of fits from PT-MCMC sampling.

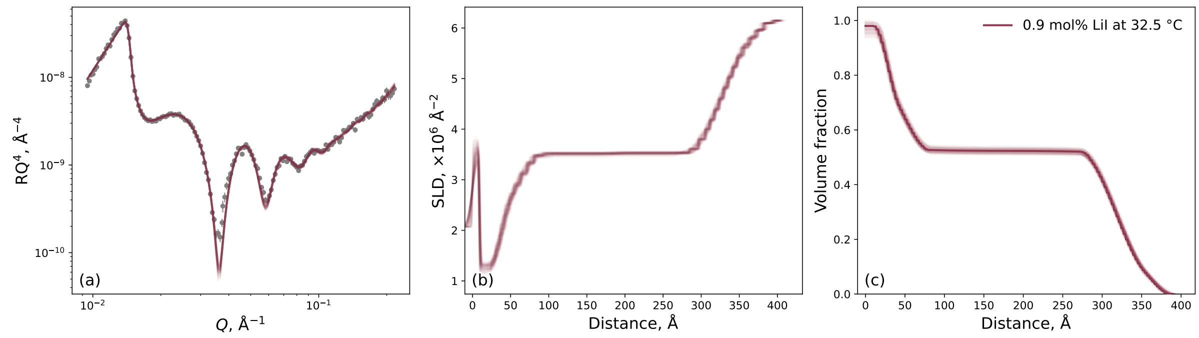

Figure S5.2xxi: (a) Reflectivity, (b) SLD and (c) polymer VF profiles of the 210 Å PNIPAM brush in a 0.9 mol% LiI electrolyte in $x_\mathrm{M} = 0.10$ at 32.5 °C with the superimposed distribution of fits from PT-MCMC sampling.

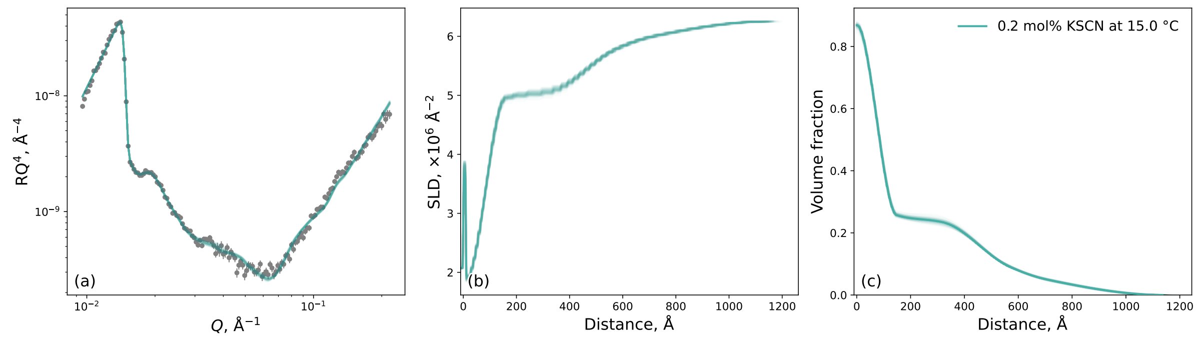

Figure S5.2xxii: (a) Reflectivity, (b) SLD and (c) polymer VF profiles of the 210 Å PNIPAM brush in a 0.2 mol% KSCN electrolyte in $x_{\mathrm{M}} = 0.10$ at 15.0 °C with the superimposed distribution of fits from PT-MCMC sampling.

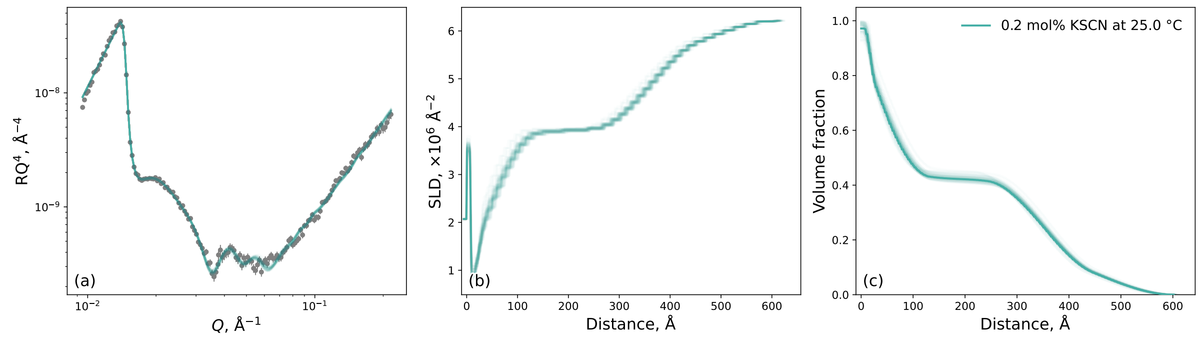

Figure S5.2xxiii: (a) Reflectivity, (b) SLD and (c) polymer VF profiles of the 210 Å PNIPAM brush in a 0.2 mol% KSCN electrolyte in $x_{\mathrm{M}} = 0.10$ at 25.0 °C with the superimposed distribution of fits from PT-MCMC sampling.

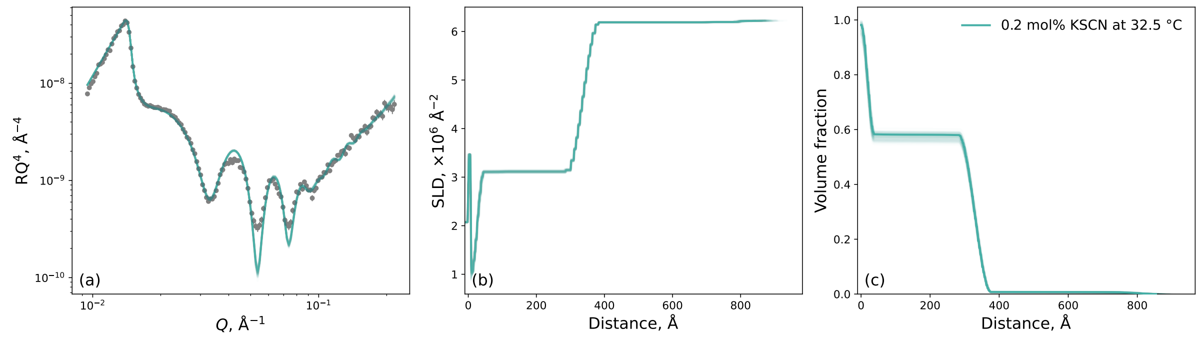

Figure S5.2xxiv: (a) Reflectivity, (b) SLD and (c) polymer VF profiles of the 210 Å PNIPAM brush in a 0.2 mol% KSCN electrolyte in $x_{\mathrm{M}} = 0.10$ at 32.5 °C with the superimposed distribution of fits from PT-MCMC sampling.

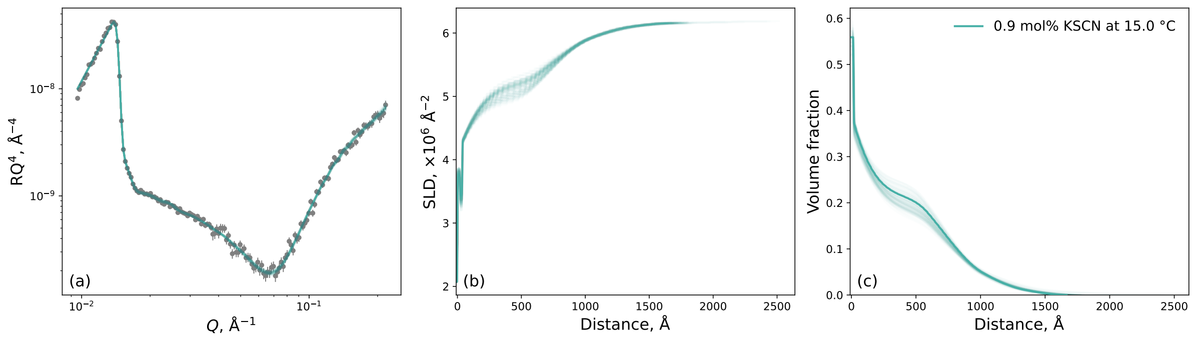

Figure S5.2xxv: (a) Reflectivity, (b) SLD and (c) polymer VF profiles of the 210 Å PNIPAM brush in a 0.9 mol% KSCN electrolyte in $x_{\mathrm{M}} = 0.10$ at 15.0 °C with the superimposed distribution of fits from PT-MCMC sampling.

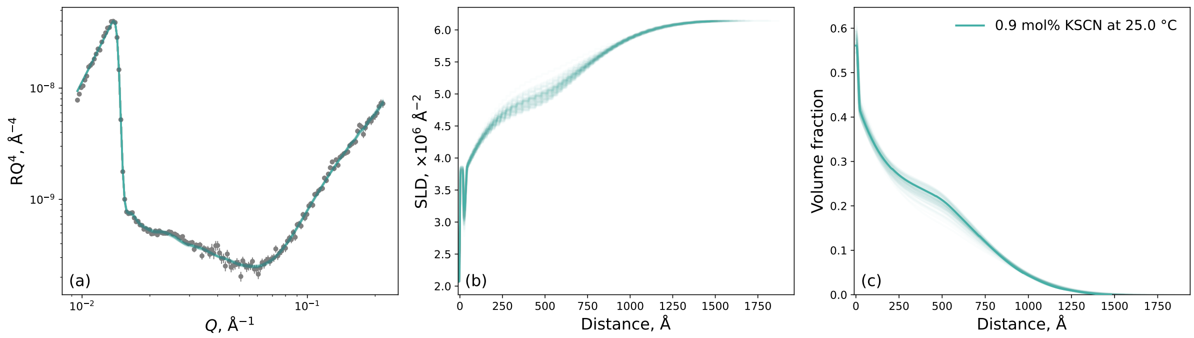

Figure S5.2xxvi: (a) Reflectivity, (b) SLD and (c) polymer VF profiles of the 210 Å PNIPAM brush in a 0.9 mol% KSCN electrolyte in $x_{\mathrm{M}} = 0.10$ at 25.0 °C with the superimposed distribution of fits from PT-MCMC sampling.

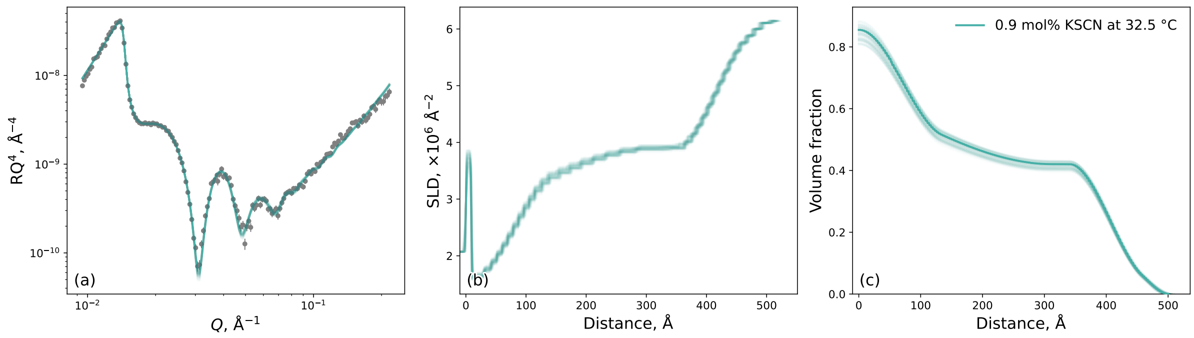

Figure S5.2xxvii: (a) Reflectivity, (b) SLD and (c) polymer VF profiles of the 210 Å PNIPAM brush in a 0.9 mol% KSCN electrolyte in $x_{\mathrm{M}} = 0.10$ at 32.5 °C with the superimposed distribution of fits from PT-MCMC sampling.

References

[1] K. P. Gregory, E. J. Wanless, G. B. Webber, V. S. Craig and A. J. Page, *Chemical Science*, 2021, **12**, 15007–15015.

[2] H. Robertson, I. J. Gresham, A. R. J. Nelson, K. P. Gregory, E. C. Johnson, J. D. Willott, S. W. Prescott, G. B. Webber and E. J. Wanless, *Langmuir*, 2024, **40**, 335–347.

[3] A. R. J. Nelson and S. W. Prescott, *Journal of Applied Crystallography*, 2019, **52**, 193–200.

[4] I. J. Gresham, T. J. Murdoch, E. C. Johnson, H. Robertson, G. B. Webber, E. J. Wanless, S. W. Prescott and A. R. J. Nelson, *Journal of Applied Crystallography*, 2021, **54**, 739–750.

[5] H. Robertson, I. J. Gresham, A. R. J. Nelson, S. W. Prescott, G. B. Webber and E. J. Wanless, *Advances in Colloid and Interface Science*, 2024, **331**, 103238.
